# Synthetic DNA spike-in standards for cross-domain absolute quantification of microbiomes by rRNA gene amplicon sequencing

**DOI:** 10.1093/ismeco/ycaf028

**Published:** 2025-02-11

**Authors:** Dieter M Tourlousse, Yuji Sekiguchi

**Affiliations:** Biomedical Research Institute, National Institute of Advanced Industrial Science and Technology, Tsukuba Central 6, 1-1-1 Higashi, Tsukuba, Ibaraki 305-8566, Japan; Biomedical Research Institute, National Institute of Advanced Industrial Science and Technology, Tsukuba Central 6, 1-1-1 Higashi, Tsukuba, Ibaraki 305-8566, Japan

**Keywords:** absolute quantification, microbiome, rRNA gene amplicon sequencing, synthetic spike-in standards, cross-domain microbiome profiling

## Abstract

Microbiome studies using high-throughput sequencing are increasingly incorporating absolute quantitative approaches to overcome the inherent limitations of relative abundances. In this study, we have designed and experimentally validated a set of 12 unique synthetic rRNA operons, which we refer to as rDNA-mimics, to serve as spike-in standards for quantitative profiling of fungal/eukaryotic and bacterial microbiomes. The rDNA-mimics consist of conserved sequence regions from natural rRNA genes to act as binding sites for common universal PCR primers, and bioinformatically designed variable regions that allow their robust identification in any microbiome sample. All constructs cover multiple rRNA operon regions commonly targeted in fungal/eukaryotic microbiome studies (SSU-V9, ITS1, ITS2, and LSU-D1D2) and two of them also include an artificial segment of the bacterial 16S rRNA gene (SSU-V4) for cross-domain application. We validated the quantitative performance of the rDNA-mimics using defined mock communities and representative environmental samples. In particular, we show that rDNA-mimics added to extracted DNA or directly to the samples prior to DNA extraction precisely reflects the total amount of fungal and/or bacterial rRNA genes in the samples. We demonstrate that this allows accurate estimation of differences in microbial loads between samples, thereby confirming that the rDNA-mimics are suitable for absolute quantitative analyses of differential microbial abundances.

## Introduction

High-throughput sequencing of marker gene amplicons is a key technique in contemporary microbiome research. A primary goal of such studies is to identify taxa associated with particular environmental conditions. This is, however, not straightforward because sequence counts alone only capture relative abundances (i.e. microbiome data are compositional [1]). Because changes in the absolute abundance of any given taxon can change the relative abundance of all other taxa in the sample, this can lead to inaccurate conclusions or obscure biologically relevant information if not adequately accounted for [2–6]. The relative nature of sequence counts also complicates the integration of microbiome profiles across different kingdoms or domains [7]. This is because each kingdom or domain requires different PCR primers for amplicon library construction, resulting in relative abundances that represent proportions of different overall compositions.

The above challenges can be mitigated by converting sequence counts to absolute units by normalizing the data to the total microbial load (i.e. the total absolute amount of microbes) in the samples. In its simplest form, this involves multiplying relative taxon abundances measured through sequencing by experimentally determined microbial loads. Techniques for measuring total microbial abundances that have been integrated with sequencing fall into two categories: (i) complementary microbial quantification methods, such as quantitative real-time PCR [6, 8], digital PCR [9], and flow cytometry [10, 11], and (ii) incorporation of quantitative standards, or spike-ins, into samples [12, 13]. While each method has its strengths and limitations [14], the use of spike-ins is particularly appealing because it eliminates the need for auxiliary measurements and is also relatively easy to incorporate into existing workflows.

Spike-in controls for microbiome applications can be whole cells [15–18] or genomic DNA [19, 20] from cultured microbes, and artificial (or synthetic) nucleic acid sequences (see references below). Artificial sequences are attractive because they can be readily designed to be different from natural sequences and to have desired properties in terms of guanine-cytosine (GC) content, length, *etc*. Given these advantages, several previous studies developed synthetic spike-in controls for both amplicon sequencing of bacterial and/or fungal communities [21–25] and metagenomics [26, 27]. For amplicon sequencing, the majority of past studies described a small number of spike-ins that are compatible with only a single, or at most a few, “universal” rRNA gene primers. To facilitate wider adoption, it would be beneficial to develop a more diverse array of spike-ins that can be used with a wider range of PCR primers targeting different rRNA genes and regions. In this regard, we have previously described a set of full-length synthetic 16S rRNA genes that are compatible with PCR primers targeting any hypervariable region [21].

In this study, we have developed 12 synthetic rRNA operon sequences to serve as spike-in DNA standards for absolute quantitative analysis of fungal/eukaryotic and bacterial abundances by high-throughput amplicon sequencing. These spike-ins, which we termed rDNA-mimics, cover multiple segments of the rRNA operon commonly targeted in fungal/eukaryotic microbiome studies. Two of them also include an artificial segment of the bacterial 16S rRNA gene for quantitative analysis across two domains. Because the rDNA-mimics are added directly to the samples and processed together with the natural sequences in the samples throughout the entire workflow, they act as competitive controls that provide robust references for normalizing read counts to the microbial load in the samples or, equivalently, for absolute quantification of the detected taxa. Here, we describe the design of rDNA-mimics and validate their suitability as internal standards for the absolute quantification of microbiomes across the two domains.

## Materials and methods

### Design of the rDNA-mimics

Following our previously described strategy [21], sequences of the rDNA-mimics were created by substituting the variable regions in natural rRNA operons with unique artificial sequences that are distinct from known sequences. To this end, artificial sequences were designed starting from a set of randomly generated 20-mers, with desired GC content and lacking homopolymers of >3 bp. The 20-mers then were progressively assembled into longer sequences, at each step screening for balanced base composition, direct and inverted repeats of >8 bp, uniform GC content (within 2.5% of the overall GC content), prohibited k-mers (such as 8-mers matching the intended PCR primers), and sequence similarity (alignments of >16 bp, as evaluated by pairwise local alignment) within and between sequences. Resultant sequences were further assessed for secondary structure formation using the M-fold server [28] and queried against NCBI’s nt/nr database by nucleotide BLAST (last verified on August 10, 2024). Natural rRNA gene sequences of *Saccharomyces cerevisiae* (strain S288C) and *Cryptococcus neoformans* (strain H99) were extracted from genome sequences available in Genbank, under accession numbers NC_001144.5 and NC_026746.1, respectively. Conserved regions in the rRNA genes were identified based on commonly used PCR primer sequences and the intervening regions were replaced by the artificial sequences. A summary of the rDNA-mimics and their sequences, with the natural conserved and artificial variable regions highlighted, are provided in Table S1 and Table S2, respectively.

### Preparation of the rDNA-mimics as linearized plasmid DNA

Full-length rDNA-mimics were chemically synthesized and cloned into plasmid vectors (pUC19) by Genscript (Tokyo, Japan). For production, plasmids were transformed into ECOS Competent *Escherichia coli* DH5α cells (Nippon Gene) and plasmid DNA was extracted from overnight cultures using a QIAprep Spin Miniprep Kit (Qiagen), both following the manufacturer’s provided instructions. Plasmid DNA was linearized using the restriction enzymes BsaI (for Sc4001, Cn4001, Sc5002, Sc5004, Sc5005, Sc5006, Cn5001, and Sc5002) or BpmI (for Sc5001, Sc5003, Sc6001, and Cn6001), using the buffers and conditions recommended by the manufacturer (New England Biolabs). Linearized plasmid DNA was purified using the Agencourt AMPure XP system (Beckman Coulter), and inspected by electrophoresis using the Agilent 2200 Tapestation system with a D5000 ScreenTape (Agilent). DNA concentrations were quantified with a high-sensitivity Quant-iT dsDNA Assay Kit (Invitrogen) using a Qubit Fluorometer 3.0 (Life Technologies). Plasmid DNA was diluted to 10 ng/μl in Tris-EDTA buffer (pH 8.0), and stored in single-use aliquots at −80°C. The full-length sequences of the rDNA-mimics were confirmed by Sanger sequencing following the procedures we described previously [21], and in all cases matched the expected sequences.

### Bacterial and fungal mock communities

The bacterial mock community consisted of near full-length 16S rRNA genes of 14 strains cloned into pUC19 plasmid vectors, as we described previously [21]. For the fungal mock community, we cloned partial rRNA operon sequences of the following strains: *Hymenoscyphus varicosporoides* NBRC 104355, *Emericella nidulans* NBRC 30063, *Schizosaccharomyces pombe* NBRC 11645, *C. neoformans* ATCC 32719, *Candida glabrata* NBRC 0622, *S. cerevisiae* NBRC 1136, *Candida tropicalis* NBRC1400, *Marasmius purpureostriatus* NBRC 30141, *Aspergillus oryzae* NBRC 100959, and *Trichoderma reesei* NBRC 31329. Purified genomic DNA of the strains was obtained from the NITE Biological Resource Center (NBRC) at the National Institute of Technology and Evaluation (NITE, Japan). For *C. neoformans*, the ZymoBIOMICS Microbial Community DNA Standard (Zymo Research) was used as source material. Partial rRNA operons were amplified by PCR using forward and reverse primers 1391f (5’-GTACACACCGCCCGTC-3′) and LR3r (5’-GGTCCGTGTTTCAAGACGG-3′), encompassing the V9 region of the small-subunit (18S) rRNA gene to the D1D2 region of the large-subunit (26S/28S) rRNA gene. PCR reactions (20 μl) contained 1× KAPA HiFi HotStart ReadyMix and 500 nM each of forward and reverse primer. Thermal cycling conditions were as follows: 3 min at 95°C, 95°C for 30 s, and 68°C for 1 min (25 cycles), and 5 min at 72°C. Amplicons were purified using the Agencourt AMPure XP system and cloned into a pUC19 plasmid vector (Nippon Gene) with the DNA Ligation Kit Ver.2.1 (Takara Bio), following the manufacturer’s instructions. Transformation of the plasmids into *E. coli* DH5α cells, linearization, and purification of plasmid DNA were performed as described earlier for the rDNA-mimics, including linearization of the plasmid DNA using BpmI for all strains, except *A. oryzae* (ScaI) and *C. neoformans* (BsaI). Sanger sequencing was performed and the resultant sequences were used as references for analysis (see below).

### Soil DNA extraction

Soil sample was collected at the National Institute of Advanced Industrial Science and Technology (Tsukuba, Japan). Extraction of DNA was performed using the FastDNA SPIN Kit for Soil (MP Biomedicals), following the manufacturer’s manual. If applicable, rDNA-mimics were added to the samples after cell lysis by bead-beating.

### Amplicon library construction and sequencing

Amplicon libraries were constructed by two-step tailed PCR, largely following Illumina’s “16S Metagenomic Sequencing Library Preparation” protocol. First-round PCR reactions (20 μl) consisted of 1× KAPA HiFi HotStart ReadyMix, 500 nM each of forward and reverse primer (see Table S3 for primer sequences and Table S4 for amplicon characteristics), and 2 μl of DNA template. The latter contained 2 ng of environmental DNA (soil DNA, spiked with rDNA-mimics before or after DNA extraction) or ~ 2 × 10^5^ total copies of the rDNA-mimics and/or mock community plasmid DNA. The same thermal cycling scheme, except for the annealing temperature, was used for all primer sets, consisting of enzyme activation for 3 min at 95°C, 20 to 25 cycles of denaturation (95°C), primer annealing, and extension (72°C), each for 30 s, and a final extension for 5 min at 72°C. Annealing temperatures for the different primer sets were as follows: SSU-V9: 50°C; ITS2: 52°C; LSU-D1D2: 57°C; ITS1: 52°C and SSU-V4: 50°C. PCR products were purified with the Agencourt AMPure XP system, using a 1:1 bead-to-sample ratio, and eluted in 25 μl of buffer. Second-round PCR reactions (20 μl) consisted of 1× KAPA HiFi HotStart ReadyMix, 2 μl each of barcoded primer (Nextera XT Index Kit v2, Illumina), and 2 μl of purified first-round PCR product. Thermal cycling conditions were as above, except for the annealing temperature (here, 55°C) and number of cycles (here, 8). After clean-up as above, the amplicons were inspected and quantified using the D1000 ScreenTape system (Agilent) and pooled at equimolar concentrations. The pooled libraries were supplemented with PhiX control DNA (up to 30%) and sequenced on an Illumina Miseq instrument using V2 chemistry (2 × 251-bp reads).

### Processing and analysis of sequence reads

Binary Base Call (BCL) files were converted to FASTQ format using Illumina’s bcl2fastq conversion software (v2.16.0.10; default parameters). Reads were additionally processed with Atropos v1.1.31 [29] to trim potentially remaining adapters, with options --aligner insert --no-default-adapters --adapter CTGTCTCTTATACACATCT --adapter2 CTGTCTCTTATACACATCT. Next, primers in the reads were identified and removed using Cutadapt v4.3 [30], using the primer-specific parameters shown in Table S5. In all cases, sequences lacking identifiable primers and undetermined bases (flags --max-n 0 --discard-untrimmed) were removed. Sequences were then further filtered based on their expected errors using DADA2’s v1.26.0 [31] filterAndTrim function (see Table S5). Reads were then denoised to construct amplicon sequence variants (ASVs), following DADA2’s common workflow, including learning the error models (function *learnErrors*) and denoising (function *dada*). Only the forward reads were used for primer sets SSU-V9, ITS1, and ITS2. For SSU-V4 and LSU-D1D2 amplicons, forward and reverse reads were denoised separately and subsequently combined using DADA2’s *mergePairs* command, with the option justConcatenate = TRUE for LSU-D1D2 amplicons. Sequence data from different sequencing runs were processed separately and then merged into a single ASV count table. For taxonomic assignment, we used DADA2’s *assignTaxonomy* function with a custom database of the sequences of the rDNA-mimics and strains in the mock communities. If applicable, multiple ASVs assigned to a given rDNA-mimic or species in the mock community were combined and their counts summed. For environmental samples, all ASVs not assigned to the rDNA-mimics were considered as environmental ASVs and their counts were summed to represent total microbial loads.

### Data analysis

Data analysis and visualization were performed in R v4.4.1 [32], via the RStudio GUI v2023.3.1.446 [33], mainly using the packages part of the “tidyverse” v2.0.0 [34], including dplyr v1.1.4 and tidyr v1.3.1 for data handling and ggplot2 v3.5.1 for plotting. If applicable, random subsampling (without replacement) of count tables was performed using vegan’s v2.6 [35] *rrarefy* function. Fitting of rectangular hyperbola was performed using the function *drm* (with fct = MM.2()) of the R package dcr v3.0 [36]. Linear regression analysis was performed using R stats’ *lm* function. Linear correlations between actual and measured values were calculated as Pearson’s correlation coefficients, calculated using R stats’ *cor* function. Differences between expected and measured values were expressed as the geometric mean of absolute fold differences [21], which is equivalent to the mean of the differences in log-transformed values.

## Results

### Design and experimental validation of the rDNA-mimics

**Figure 1:**
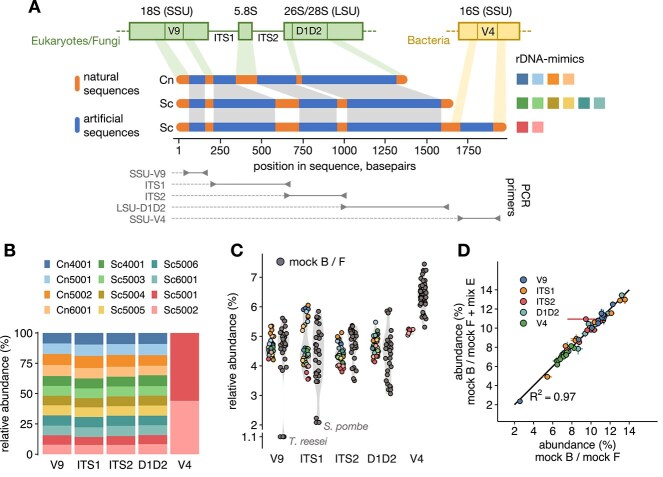
**(A)** Overview of the rDNA-mimics. The upper part of the schematic shows the organization of the fungal/eukaryotic rRNA operon along with the partial bacterial 16S rRNA gene (V4 hypervariable region) incorporated in two of the rDNA-mimics. The middle part shows the layout of the rDNA-mimics, consisting of artificial sequences flanked by conserved sequences of the natural rRNA genes of the yeasts *S. cerevisiae* (Sc) and *C. neoformans* (Cn). Colored squares on the right correspond to the legend in panel B. The lower part indicates the location of the PCR primers used for experimental evaluation of the rDNA-mimics. **(B)** Testing of the rDNA-mimics with an equimolar formulation of all 12 rDNA-mimics (mix E). Stacked bar charts of the relative abundances of the rDNA-mimics, averaged over three technical replicates, for amplicon libraries generated with the primer sets shown on the *x*-axis. **(C)** Testing of the rDNA-mimics with mix E added to an even fungal or bacterial mock community. Each symbol shows an individual data point from three technical replicates. Symbols for the rDNA-mimics are colored as in panel B and data for all species in the mock communities are shown as dark grey circles. **(D)** Scatter plot of species abundances for the fungal (mock F) or bacterial (mock B) mock community + mix E samples (*y*-axis) against the mock community-only samples (*x*-axis). Data are shown as the mean (circles) and SD (error bars) of three technical replicates, with fill colors indicating the primer set. The solid line represents the 1:1 diagonal and the corresponding R^2^ value is indicated.

We designed 12 unique synthetic rRNA operons (rDNA-mimics) starting from the sequences of the rRNA operons of two fungi, namely the type strains of the yeasts *S. cerevisiae* (Sc, strain S288C) and *C. neoformans* (Cn, strain H99). Following the approach outlined in our previous work [21], the rDNA-mimics were generated by replacing the variable regions of the Sc and Cn rRNA genes with bioinformatically designed artificial sequences that are fully distinct from known sequences in the NCBI nr/nt database. This layout allows the rDNA-mimics to be amplified by PCR primers targeting the conserved natural sequences and robustly identified in any microbiome sample based on their artificial sequence regions. To facilitate PCR amplification and sequencing, the artificial sequences were designed with balanced nucleotide composition and uniform GC content (40%, 50%, or 60%; Fig. S1) and screened to avoid homopolymers, repeats, similarity within and between sequences, and secondary structures. As shown in Fig. 1A, the rDNA-mimics contain conserved regions flanking the eukaryotic/fungal V9 hypervariable region of the small subunit (SSU) rRNA gene, the fungal internal transcribed spacers (ITS1 and ITS2), and the D1/D2 domain of the fungal large subunit (LSU) rRNA gene. These regions were selected because they represent widely used marker loci for characterizing eukaryotic (SSU-V9) and fungal (ITS1, ITS2, and LSU-D1D2) microbial communities [37–39]. For cross-domain application, two Sc-based rDNA-mimics also contain artificial versions of the V4 hypervariable region of the bacterial 16S rRNA gene, a commonly targeted region in microbiome studies, including the Earth Microbiome Project [40], flanked by conserved sequences from *E. coli* [21]. The rDNA-mimics, which had a length of 1320 to 1919 bp (Table S1**,** see Table S2 for marked-up sequences), were chemically synthesized, inserted into a plasmid vector for propagation, and used in the form of linearized plasmid DNA.

For initial experimental testing, we analyzed an equimolar mixture of all 12 rDNA-mimics (referred to as mix E) using the eukaryotic/fungal primer sets SSU-V9, ITS1, ITS2, and LSU-D1D2, as well as the bacterial primer set SSU-V4 (Fig. 1A, see Table S3 for primer sequences). After sequencing on an Illumina MiSeq instrument and denoising the sequences with DADA2, the relative abundances of all rDNA-mimics were comparable across all tested fungal/eukaryotic primer sets (Fig. 1B). Within each primer set, deviations from the expected abundances, expressed as the geometric mean of absolute fold differences (gmAFD, averaged across rDNA-mimics), ranged from 1.05 for the SSU-V9 primer set to 1.10 for the ITS1 primer set. The slightly higher deviation between expected and measured abundances for the ITS1 primer set was largely due to the differences in the abundance of the Cn-based rDNA-mimics (9.4 ± 1.0%, mean ± SD) compared to the Sc-based rDNA-mimics (7.8 ± 0.7%). This discrepancy may be explained by their non-identical PCR primer binding sites and/or differences in amplicon sizes (Table S4) as both factors have been shown to affect detection efficiency in the context of amplicon sequencing [41, 42]. For the SSU-V4 primer set, both rDNA-mimics Sc5001 and Sc5002 were also detected with nearly equal abundance (Fig. 1B). These results confirmed that the rDNA-mimics can be effectively and uniformly amplified by PCR using common eukaryotic/fungal and bacterial primers targeting different regions of the rRNA operon.

We next tested whether the rDNA-mimics exhibited comparable detection efficiencies to diverse natural rRNA gene sequences. For this purpose, we combined mix E with mock communities consisting of plasmid DNA with cloned rRNA genes from 10 fungal or 14 bacterial species, such that all sequences were equally abundant in the combined mixtures. For the fungal primer sets and fungal mock + mix E, we found that the abundance of most rDNA-mimics was within the range of the abundances of the species in the mock community (Fig. 1C). Two species, namely *T. reesei* and *S. pombe*, yielded lower abundances for SSU-V9 and ITS1 primer sets, respectively. This could be explained by primer mismatches between the SSU-V9 primers and *T. reesei* (Table S4), whereas the ITS1 primers perfectly matched *S. pombe*, and other unidentified factors were thus likely responsible for its moderate underestimation. For the bacterial SSU-V4 primer set, the abundance of the two rDNA-mimics was slightly lower than the abundance of the species in the bacterial mock (Fig. 1C). Differences were however relatively small (1.2-fold compared to the mean abundance of the mock community members), and may be due to factors such as PCR and/or sequencing bias or inaccuracies introduced during sample preparation. These results showed that the detection efficiency of the rDNA-mimics is largely comparable to that of diverse natural rRNA genes, hence confirming their suitability as internal standards for quantitative microbiome analyses. Furthermore, the rDNA-mimics did not interfere with the detection of the mock community members, as shown by comparing the fungal or bacterial mock + mix E samples to the mock community-only samples (Fig. 1D).

### Quantitative performance of rDNA-mimics added to complex environmental DNA

To further validate the rDNA-mimics, we evaluated their quantitative performance by spiking them at varying levels (as absolute copy numbers) into DNA extracted from soil, as a representative diverse environmental sample. Specifically, we added decreasing amounts of mix E, between approximately 2 × 10^2^ and 2 × 10^6^ total copies, to a fixed amount of soil DNA (2 ng), and constructed amplicon libraries using all the above primer sets. The abundances of the rDNA-mimics were determined after denoising of the sequence data with DADA2; here, ASVs not assigned to the rDNA-mimics were considered as environmental taxa and their counts were summed to represent the total amount of soil-derived sequences in the samples. For the SSU-V4 primer set, the Sc5001 and Sc5002 rDNA-mimics yielded at most 25 reads per sample at the lowest spike-in level; these data were thus excluded prior to analysis. Similarly, data for the fungal primer sets with the highest spike-in level were omitted because virtually all reads (98.7 ± 0.5%) belonged to the rDNA-mimics. Across the remaining samples, ASV count tables contained >40 000 reads and were analyzed without subsampling, unless otherwise noted.

**Figure 2:**
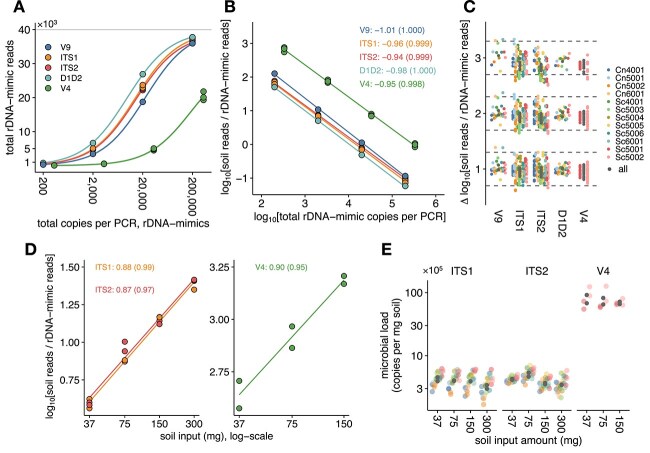
**(A)** Relationship between total rDNA-mimic reads (i.e. summed across rDNA-mimics) and their spike-in levels (i.e. total copies per PCR). Each symbol represents an individual data point, colored by primer set as indicated in the legend. Colored lines show the fitted rectangular hyperbola for each primer set. The grey horizontal line indicates the subsampling depth (i.e. 40 000 reads per sample). **(B)** Relationship between ALR-transformed soil reads and rDNA-mimic spike-in levels. Solid lines show the best-fitting linear regression line (log–log scale); slopes and R^2^ values (within parentheses) are added as text labels, colored as in panel A. **(C)** Conconcordance between actual and measured differences in ALR-transformed soil read counts. For this analysis, we considered all pairs of samples with different rDNA-mimic levels, resulting in expected values of log_10_(10), log_10_(100) and log_10_(1000), as indicated on the *y*-axis. For each log-fold difference on the *y*-axis, dashed horizontal lines show two-fold differences between actual and measured values. Symbols represent individual data points, colored by the rDNA-mimic used as the basis for ALR transformation. Grey symbols show the data using the aggregated rDNA-mimic reads for ALR transformation. **(D)** Relationship between ALR-transformed microbial read counts (*y*-axis), based on the aggregated rDNA-mimic reads, and the amount of soil subjected to DNA extraction (*x*-axis), for samples supplemented with a constant amount of an equimolar formulation of the rDNA-mimics. Each symbol represents a data point for three primer sets (ITS1, ITS2, and SSU-V4, colored as in panel A). Solid lines show the best-fit log–log linear regression line, with slopes and R^2^ values (within parentheses) shown as text labels. **(E)** Normalized microbial loads (i.e. estimated copies per mg of soil) as determined using each of the rDNA-mimics as the basis for quantification. Each symbol represents a different data point, colored as in panel C. Estimates based on the summed rDNA-mimic counts are shown in dark grey.

For all primer sets, the number of reads (after random subsampling to equal depth) assigned to the rDNA-mimics increased consistently with higher spike-in levels (Fig. 2A). Non-linear regression further showed that the data for each primer set followed the expected rectangular hyperbolic trend when plotting rDNA-mimic read counts, after subsampling, against spike-in levels. This demonstrated that the observed read counts precisely reflected the abundance of the rDNA-mimics, relative to the total microbial load, in the samples over multiple orders of magnitude.

To further illustrate this, we also analyzed the ratio of environmental (numerator) to rDNA-mimic (denominator) read counts. After applying a log transformation, these ratios are known as additive log ratios (ALR) in compositional data analysis [43]. Equivalently, they also represent scaling factors to normalize read counts by total microbial loads or to convert microbial read counts to absolute quantities (e.g. copy number per unit amount of sample). Plotting the ALR-transformed microbial counts against spike-in levels on a double logarithmic scale should here, by design, result in curves described by a linear trend line with a slope of −1 (see **Supplementary Note 1** for mathematical expressions). Considering total rDNA-mimic reads as the basis for ALR transformation, the best-fit linear regression slopes of the curves closely matched the expected value for all primer sets (−0.97 ± 0.03), with high coefficients of determination (R^2^ > 0.99; Fig. 2B). Similarly, when using the reads for each rDNA-mimic separately for ALR transformation, the slopes remained close to −1 (−0.98 ± 0.05, mean ± SD across primer sets and rDNA-mimics), again with high R^2^ values (Fig. S2). These data showed that the rDNA-mimics are suitable as references for ALR transformation or converting microbial read counts to absolute scale.

Since differential abundance analyses in microbiome research primarily focus on comparing absolute abundances between samples [44], we also calculated the differences in ALR-transformed counts between all pairs of samples, and compared them to the values theoretically expected by design. Based on the experimental design in which varying levels of rDNA-mimics (as absolute copy numbers) were added to a fixed amount of soil DNA, differences in ALR-transformed soil-derived reads between samples are expected to be equal to the log-fold differences in rDNA-mimic levels between samples, up to a minus sign, as shown in **Supplementary Note 1**. Since the rDNA-mimics were added at four different levels in 10-fold dilution steps, this thus amounted to expected values of log_10_(10), log_10_(100) and log_10_(1000). As anticipated from the near-unity slopes of the above dose–response curves, we observed good to excellent agreement between expected and actual values (Fig. 2C). Across all data, measured values deviated 1.29-fold (gmAFD; interquartile range [IQR]: 1.09–1.45) from the expected values, with 79.1% of comparisons showing differences of less than 1.5-fold. When averaged separately for each rDNA-mimic and primer set, gmAFDs ranged from 1.16 for primer set LSU-D1D2 to 1.35 for ITS2, and from 1.17 for rDNA-mimic Cn4001 to 1.42 for Sc5003. These data confirmed that rDNA-mimics accurately capture differences in microbial loads between samples, thus validating their suitability for absolute differential abundance analyses.

### Determination of microbial loads using rDNA-mimics spiked before DNA extraction

We next evaluated the effectiveness of the rDNA-mimics when incorporated directly into samples before DNA extraction. For this purpose, we added a constant amount of mix E, approximately 4 × 10^6^ total rDNA-mimic copies, to varying amounts of soil (37, 75, 150, and 300 mg). Here, we note that samples were spiked after cell lysis to avoid potential shearing of the rDNA-mimics. Following DNA extraction, amplicon libraries were generated using the fungal primer sets ITS1 and ITS2, and the bacterial primer set SSU-V4. The 300-mg soil sample yielded fewer than 10 reads for the rDNA-mimics with primer set SSU-V4 and was therefore excluded from the analysis. For all remaining samples, ASV count tables contained >60 000 reads per sample.

As expected, the number of reads (after random subsampling to equal depth) assigned to the rDNA-mimics decreased monotonically with increasing soil biomass, again following the theoretically expected trend (Fig. S3). As with the previous experiment, the linearity of the dose–response curves was assessed by plotting the ratio of soil to rDNA-mimic read counts against soil biomass on a log–log scale. As shown in Fig. 2D, the resultant curves based on the aggregated rDNA-mimic read counts demonstrated good to excellent linearity for each primer set, with slopes close to 0.9 and R^2^ values of >0.95. Similar results were observed for curves calculated for each rDNA-mimic individually, with slopes and R^2^ values of 0.89 ± 0.08 and 0.95 ± 0.04, respectively (Fig. S4).

In view of these results, the estimated microbial loads for both eukaryotes/fungi and bacteria, calculated based on the defined amount of each of the rDNA-mimics added to the samples, showed a strong correlation with soil biomass (Fig. S5), with Pearson’s correlation coefficients of 0.95 ± 0.04 (IQR: 0.95–0.98). Furthermore, normalized microbial loads (i.e. copy numbers per mg of soil) for both fungi and bacteria remained largely unaffected by soil DNA input amount (Fig. 2E), with an average coefficient of variation of less than 20% and an interquartile range of 14.7% to 21.0%. Collectively, these data confirmed that rDNA-mimics added prior to DNA extraction can provide precise estimates of total microbial load across the two domains.

### Demonstration of staggered rDNA-mimic mixtures and quantification of bacterial-to-fungal ratios

As noted earlier, and unlike most previous studies, we designed a set of multiple spike-ins to offer greater flexibility in their application. As an example, and to further validate the rDNA-mimics, we created a staggered pool containing the eight rDNA-mimics with a GC content of 50% (see Table S1). Using a staggered mixture of rDNA-mimics provides a straightforward way of assessing data quality based on the linearity of the standard curves for each sample [21]. Here, we applied this mixture for quantitative analyses of a series of samples with varying amounts of fungal and bacterial mock community. This allowed us to additionally assess the performance of the rDNA-mimics for determining bacterial-to-fungal (BF) microbial load ratios. As shown in Fig. S6, the samples, designated mixBFa through mixBFg, contained a fixed amount of bacterial plus fungal mock community and captured BF ratios between 40 and 0.025. The rDNA-mimic mixture was added to the samples at a fixed level in absolute units, with the amount of each of the rDNA-mimics ranging from 200 (Sc5006) to 2.5 × 10^4^ (Sc5003) copies per PCR reaction. Amplicon libraries were constructed using the fungal primer sets ITS1 and ITS2 and the bacterial primer set SSU-V4, and were quantified based on DADA2-denoised sequences. Across samples, >36 000 reads per sample (IQR: 65149–107 535) were retained in the annotated ASV count tables.

**Figure 3:**
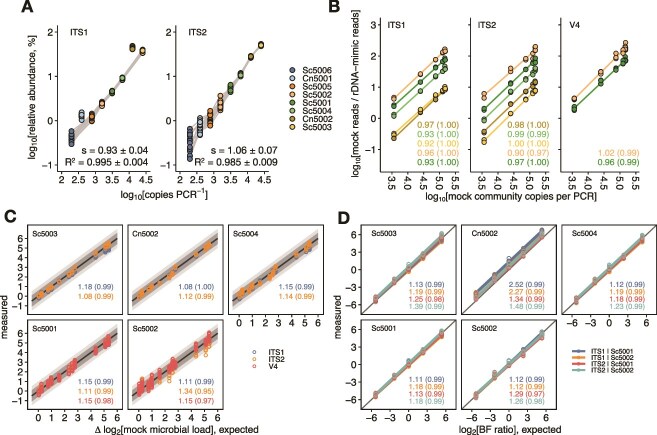
**(A)** Relationship between rDNA-mimic abundance, expressed as the percentage of reads within the subcomposition of the rDNA-mimics, and spike-in levels (i.e. copies per PCR) for the fungal primer sets ITS1 and ITS2. Symbols represent individual data points and are colored by rDNA-mimic, as the legend indicates. Grey lines connect data from individual samples. Slopes (s) and coefficients of determination (R^2^) represent the mean ± SD across samples. Note that for primer set ITS1, the Cn-based rDNA-mimics were not included in the linear regression model. **(B)** ALR-transformed mock community reads plotted against their absolute amount in mixtures mixBFa through mixBFg (see Fig. S6 for mixture formulations). Slopes and R^2^ values (within parentheses) of the regression models are shown as text labels. **(C)** Concordance between actual and measured differences in microbial loads between pairs of samples. Each symbol represents a pair of samples, colored by primer set as the legend indicates. Facet labels show the rDNA-mimic used as the basis for ALR transformation. Note that only positive expected differences are shown given the symmetry of the data. Solid back lines show the 1:1 diagonal (perfect agreement) and light and dark grey ribbons show 1.5 and 2-fold differences between expected and measured values, respectively. Text labels indicate average absolute fold-differences between actual and measured values and Pearson’s correlation coefficients (within parentheses). **(D)** Concordance between measured and actual ratios of absolute bacterial and fungal microbial loads (i.e. the BF ratio). Facet labels and symbol/line colors reflect the rDNA-mimic used for calculating fungal and bacterial loads, respectively. Average absolute fold-differences between measured and actual BF values are shown as text labels, along with Pearson’s correlation coefficients (calculated on a linear scale, within parentheses).

As alluded to above, we assessed the linearity of the measurements by plotting the number of reads, normalized within the sub-composition of the rDNA-mimics, for each of the rDNA-mimics against their copy number (Fig. 3A, see also Fig. S7). Consistent with the results of pure mix E described above, the two Cn-based rDNA-mimics exhibited noticeably higher abundances than the Sc-based controls for the ITS1 primer set. Focusing only on the Sc-based rDNA-mimics for primer set ITS1, slopes of the log–log linear regression lines were close to unity for all samples (0.93 ± 0.04). For primer set ITS2, the slopes were also close to the expected value (1.06 ± 0.07), although the variance in rDNA-mimic abundances between samples was higher than for the ITS1 primer set. As a whole, the near-unity slopes and high R^2^ values demonstrated the consistency of quantification across rDNA-mimics and concentrations, and also verified that amplicon library preparation, sequencing, and bioinformatics analyses were successfully performed. This showcased the utility of staggered rDNA-mimic pools for quality control of the quantitative performance of the entire measurement, from PCR amplification to bioinformatics analysis.

Similar to the analysis of the environmental samples described earlier, we again calculated the ratio of the aggregated read counts for the mock communities, representing the total amount of microbial rRNA operons in the samples, to the rDNA-mimic read counts to assess how well these reflected the microbial loads in the samples. For this analysis, we focused on the more abundant rDNA-mimics (i.e. Sc5003, Cn5002, Sc5004) and the two cross-domain rDNA-mimics (Sc5001 and Sc5002). As depicted in Fig. 3B, plotting this ratio against mock community loads on a log–log scale demonstrated that the rDNA-mimics precisely captured the microbial loads in the samples, as shown by the near-unity slopes (0.96 ± 0.04, across rDNA-mimics and primer sets).

Based on these results, the estimated microbial loads were strongly correlated with the actual values, with Pearson’s correlation coefficients exceeding 0.99 (Fig. S8). Still, we found that the estimated microbial loads deviated several-fold from the expected values. This bias was however consistent across samples for a given rDNA-mimic and thus canceled out when calculating differential microbial loads. This is demonstrated in Fig. 3C by comparing actual and measured differences in microbial loads between all sample pairs. Across all data, the measured values differed 1.13-fold (gmAFD; IQR: 1.04–1.18) from the actual values. Considering individual rDNA-mimics, gmAFDs varied between 1.05× for bacterial loads estimated using primer set SSU-V4 and Sc5001 to 1.34× for fungal loads evaluated with the ITS2 primer and Sc5002. Further, because total microbial loads represent a fixed scaling factor applied uniformly to all taxa in a given sample, differential abundances of individual species in the mock community were also accurately estimated (Fig. S9), with deviations between actual and expected values of 1.17 (gmAFD, averaged across species and rDNA-mimics), 1.28 and 1.21 for the ITS1, ITS2, and SSU-V4 primer sets, respectively. Further, the accuracy of the differential abundance estimates were largely comparable across species, despite species-dependent bias when considering their (relative) abundances on a per-sample basis, especially for the fungal primer sets (Fig. S10).

Finally, for cross-domain quantification, we calculated the ratio of the estimated absolute loads of bacteria to fungi and compared these to the actual BF ratios. Fungal abundances were calculated based each of the rDNA-mimics individually, whereas bacterial abundances were determined using Sc5001 and Sc5002. We then calculated BF ratios based on Sc5001 and Sc5002, which capture both fungal and bacterial in a single construct, as well as every combination of Sc5001/Sc5002 and the other fungal rDNA-mimics (i.e. Sc5003, Cn5002, Sc5004). As shown in Fig. 3D, measured BF ratios were strongly linearly correlated with actual values, with Pearson’s correlation coefficients of >0.99. Furthermore, for most rDNA-mimics, the deviation between expected and measured values was small, with an interquartile range for the gmAFD of 1.13 to 1.31. Here, we also found that estimates derived from the constructs Sc5001 and Sc5002 showed deviations from expected values that were largely comparable to those obtained from other rDNA-mimic pairs (1.17 ± 0.06 versus 1.19 ± 0.08, excluding estimates involving Cn5002). Finally, due to the strong linear relationship between expected and measured BF ratios within samples, differences in BF ratios between pairs of samples were highly accurate (Fig. S11).

## Discussion

We have introduced a series of synthetic rRNA operon constructs (rDNA-mimics) to serve as spike-in standards for quantitative analysis of eukaryotic/fungal and bacterial microbiomes. Unlike similar reagents developed in previous studies [e.g. 23, 24], our rDNA-mimics span multiple genes and regions of the eukaryotic/fungal rRNA operon. This makes them compatible with a broader range of PCR primers commonly used in eukaryotic/fungal microbiome studies, thus facilitating their wider adoption. Compared to the work of Wang et al. [45], which described five synthetic standards compatible with the bacterial 336F/806R and fungal ITS3/ITS4 primers, the rDNA-mimics described here offer greater flexibility in PCR primer selection. This advantage is due to the inclusion of longer conserved sequence regions, and not only the primer binding sites as in Wang’s constructs.

We demonstrated how rDNA-mimics, when added to extracted DNA or directly to samples prior to DNA extraction, can be reliably enumerated using different PCR primer sets and precisely reflect total fungal and bacterial loads, as total rRNA gene/operon copy numbers, in the samples. Although the mock community results showed discrepancies between actual and estimated microbial loads, this bias was generally consistent across samples and thus canceled out when comparing the resulting absolute-scale abundances between samples. We here note that rDNA-mimics, like any spike-in control, are subject to the same biases as the natural sequences in the samples. Therefore, the inferred microbial loads should better capture the microbial community that is subjected to analysis by sequencing, compared to techniques such as flow cytometry. As suggested by McClaren et al. [46] based on theoretical considerations, this should lead to more accurate estimates of fold differences in taxon abundances between samples and consequently to more reliable differential abundance analysis.

A key feature of the rDNA-mimics is their ability to assess absolute abundances of both eukaryotic/fungal and bacterial microbial taxa, thereby making the taxonomic profiles amenable to integrated analysis. Interactions between fungi and bacteria are thought to play a critical role in the functioning of a wide range of ecosystems [47], and the rDNA-mimics are therefore expected to enable more detailed investigations based on cross-domain amplicon sequencing studies. This also includes the estimation of absolute abundance ratios of fungi and bacteria—a key metric for understanding and managing soil ecosystems [48]. Although we found only relatively small differences when comparing cross-domain abundance ratios (more specifically, BF ratios) calculated using pairs of rDNA-mimics, constructs with both fungal and bacterial rRNA genes (such as Sc5001 and Sc5002) are particularly advantageous for this purpose. They simplify handling by requiring only a single rDNA-mimic per sample and their BF ratios are perfectly calibrated.

The availability of 12 unique sequences allows flexibility and enhances the versatility of the rDNA-mimics, beyond absolute quantification. Specifically, following our previous work using synthetic 16S rRNA gene spike-in controls [21], rDNA-mimics provide a useful tool for quality control and assessment of potential bias throughout the entire measurement workflow, from PCR amplification to sequencing and bioinformatics. For instance, we have shown here how staggered mixtures can be used to evaluate quantitative performance based on the consistency/linearity of measurements across different rDNA-mimics and concentrations. As an example of bias, we observed that the Cn-based rDNA-mimics were consistently detected at higher abundances than the Sc-based rDNA-mimics when using the ITS1 primer; this was presumed to be related to their differences in PCR primer binding sites or amplicon lengths. Given the considerable variation in the size of the ITS1 region among fungi [42], this may lead to significant inaccuracies in observed fungal profiles. To address this, the inclusion of rDNA-mimics in the samples, albeit preferably with a broader size range, could provide adjustment factors to correct for biases introduced by differences in amplicon length.

While incorporating the rDNA-mimics in existing workflows is relatively straightforward, users should keep several points in mind. First, a preliminary estimate of the expected microbial load in the samples may be required to set the appropriate amount of rDNA-mimics to incorporate into the samples, such that a sufficient number of rDNA-mimic reads are obtained for robust quantification without sacrificing too much sequencing capacity. For simplicity, this could be done by quantifying total microbial loads in representative samples using quantitative PCR, preferably with the same primers as used to prepare the amplicon libraries for sequencing. Such a two-tiered approach may be justified given the benefits of competitive PCR assays, which underlies the rDNA-mimics, to be more accurate in the presence of e.g. PCR inhibitors [49] or other types of procedural bias. Second, because the cross-domain rDNA-mimics have a fixed one-to-one ratio of fungal to bacterial abundances, they may be more difficult to apply to samples with widely varying fungal and bacterial loads. In this case, it may be advisable to use the here-described fungi-only rDNA-mimics in conjunction with the full-length synthetic rRNA genes we described previously [21]. This would not only allow users to independently vary the amount of fungal and bacterial rDNA-mimics added to the samples but also enable other 16S rRNA gene regions to be targeted and generate multi-point dose–response curves for quality assessment.

## Conclusion

The benefits of absolute quantitative microbiome profiling are increasingly recognized in the microbiome field, and this has spurred efforts to establish frameworks for absolute quantitative analysis. While a number of statistical techniques have been developed for differential abundance analysis of compositional microbiome data, a recent benchmarking study advocated the use of experimental quantitative methods [50]. Such techniques are expected to improve accuracy, facilitate statistical analysis, and provide more detailed biological insights. The herein-described rDNA-mimics thus provide a valuable resource for future microbiome studies.

## Supplementary Material

Tourlousse_supplement_ycaf028

## Data Availability

Full-length sequences of the rDNA-mimics are available in the DDBJ/GenBank/EMBL nucleotide sequence database under accession numbers PQ159721–PQ159732. Raw Illumina sequence reads have been deposited in NCBI’s Sequence Read Archive under BioProject PRJNA1100950 (https://www.ncbi.nlm.nih.gov/bioproject/?term=PRJNA1100950). All source data and code are available in the Zenodo repository under DOI 10.5281/zenodo.14592209 (https://zenodo.org/records/14592209).
