# Correction to: Synthetic DNA spike-in standards for cross-domain absolute quantification of microbiomes by rRNA gene amplicon sequencing

**DOI:** 10.1093/ismeco/ycaf112

**Published:** 2026-02-13

This is a correction to: Dieter M Tourlousse, Yuji Sekiguchi, Synthetic DNA spike-in standards for cross-domain absolute quantification of microbiomes by rRNA gene amplicon sequencing, *ISME Communications*, Volume 5, Issue 1, January 2025, ycaf028, 10.1093/ismeco/ycaf028

The online version of this paper has been updated to clarify that the original publication date for the paper was 11 February 2025 (not 17 March 2025).

