# Supplementary material for: Synthetic DNA spike-in standards for cross-domain absolute quantification of microbiomes by rRNA gene amplicon sequencing
Source: ISME Commun. 2025 Feb 11;5(1):ycaf028. doi: 10.1093/ismeco/ycaf028 (PMC11912825; doi:10.1093/ismeco/ycaf028)
Supplement: Tourlousse_supplement_ycaf028 [file tourlousse_supplement_ycaf028.pdf]

Supplementary notes

Note 1

This note provides mathematical expressions to support the results presented in the main text.

To derive the expressions, we start from the notion that the ratio of the absolute abundances of two taxa (A_i and A_j) in a given sample is equal to the ratio of their read counts (R_i and R_j) in the corresponding sequencing data, that is:

$$\frac{R_i}{R_j} = \frac{A_i}{A_j}$$

Taking the logarithm on both sides then yields:

$$\log\frac{R_i}{R_j} = -\log A_j + \log A_i$$

In case *j* represents is interpreted as a fixed reference (here, rDNA-mimics), the left-hand side of the equation can then be recognized as the additive log ratio (ALR) transformation, more specifically the ALR-transformed read counts of ith taxon with respect to the fixed reference (renamed as *r* below for clarity):

$$\log\frac{R_i}{R_r} = ALR_{i,r} = -\log A_r + \log A_i$$

For an experiment in which varying amounts of the reference are added to a constant amount of microbial load, it can thus be seen that plotting the ALR-transformed taxon read counts as a function of the amount of reference added yields a linear model with a slope of -1. This is the basis for the data presented in Fig. 2B.

In similar fashion, it can be seen that the difference in ALR-transformed read counts for any given taxon *i* to rDNA-mimic *r* is equal to the logarithm of the ratio of the absolute amounts of the rDNA-mimic added to the two samples (here named *s* and *t*), up to a minus sign.

$$\mathrm{ALR}_{i,r,s} = -\log A_{r,s} + \log A_{i,s}$$

$$\mathrm{ALR}_{i,r,t} = -\log A_{r,t} + \log A_{i,t}$$

$$ALR_{i,r,s} - ALR_{i,r,t} = \log A_{r,t} - \log A_{r,s} + \log A_{i,s} - \log A_{i,t} = \log\frac{A_{r,t}}{A_{r,s}} = -\log\frac{A_{r,s}}{A_{r,t}}$$

where $\log A_{i,s}$ is equal to $\log_{Ai,t}$ by design and thus cancels out. This is the basis for the data presented in Fig. 2C.

Supplementary figures

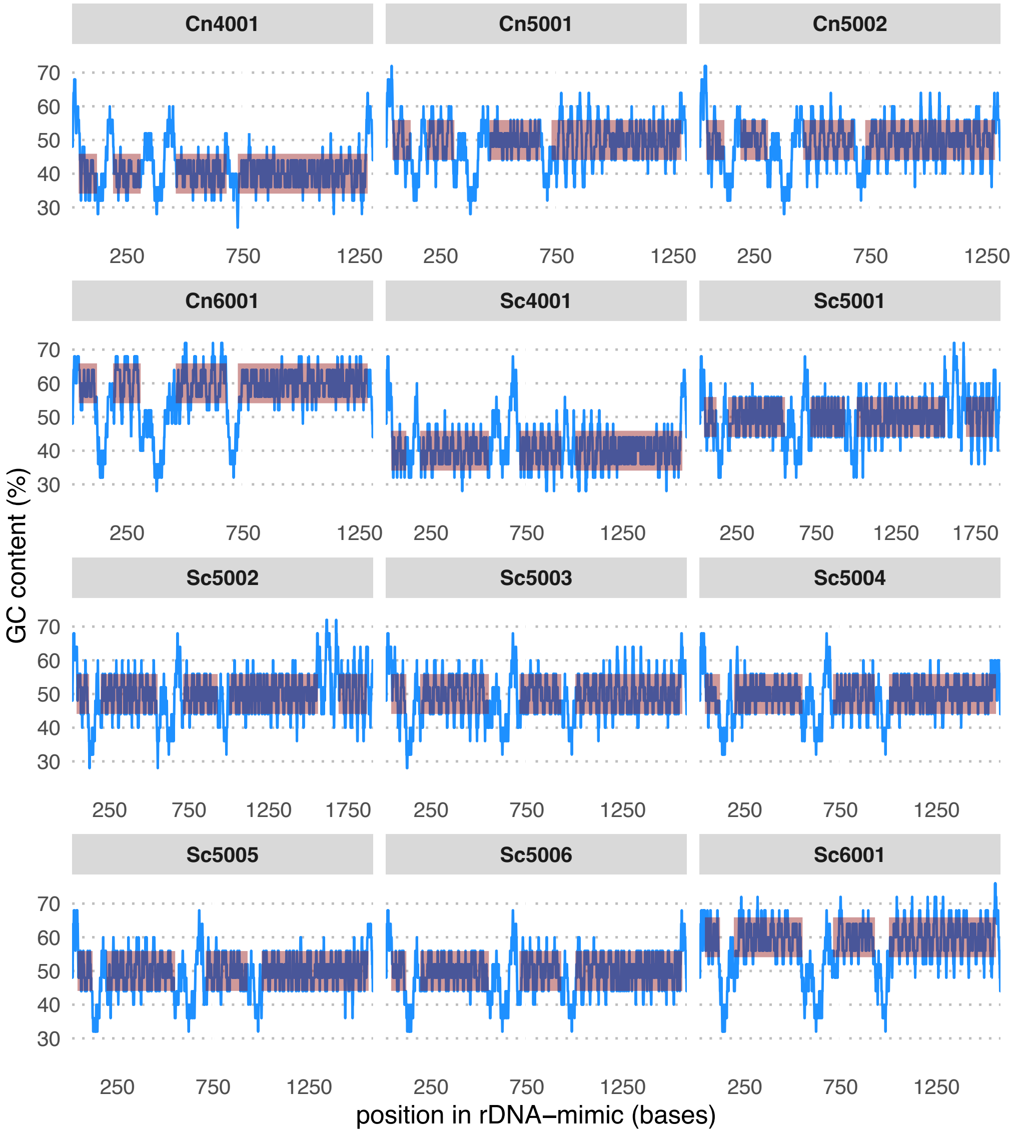

Fig. S1. Guanine-cytosine (GC) content, calculated over 25 base pair moving windows, of the rDNA-mimics. Segments highlighted by red ribbons represent the artificial sequence regions.

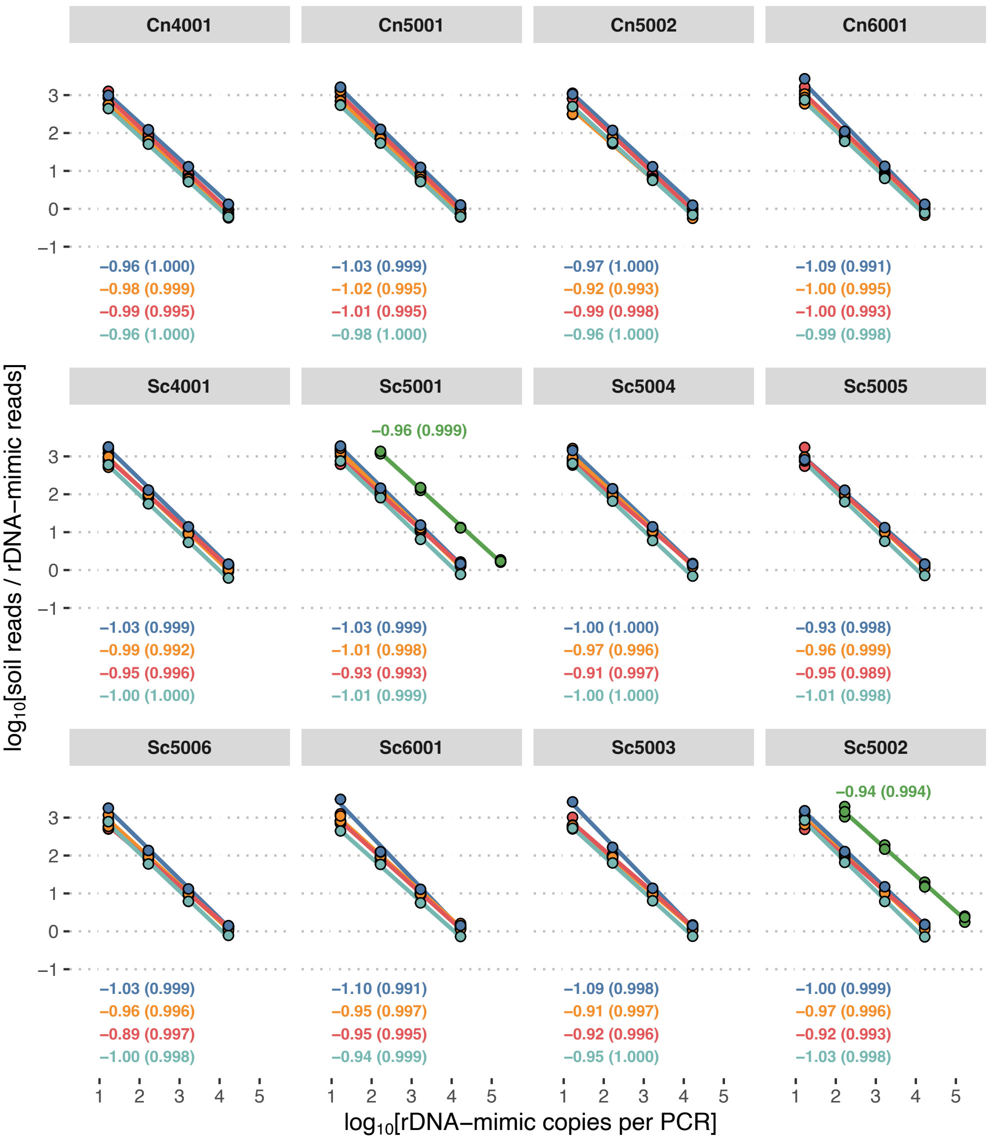

Fig. S2. Scatter plot of $\log_{10}$-transformed ratios of soil reads to rDNA-mimic reads (that is, ALR-transformed soil reads, *y*-axis) plotted against rDNA-mimic copy numbers ($\log_{10}$-transformed, *x*-axis) for samples in which varying amounts of rDNA-mimics was added to a fixed amount of soil DNA. Facet labels indicate the rDNA-mimic set as the basis for ALR transformation. Symbols represent individual data points, colored by primer set (blue: V9; orange: ITS1; red: ITS2; light teal: D1D2; green: V4). Solid lines show the best-fitting linear regression (log-log scale) models, with the corresponding slopes and coefficients of determination (within parentheses) shown as text labels.

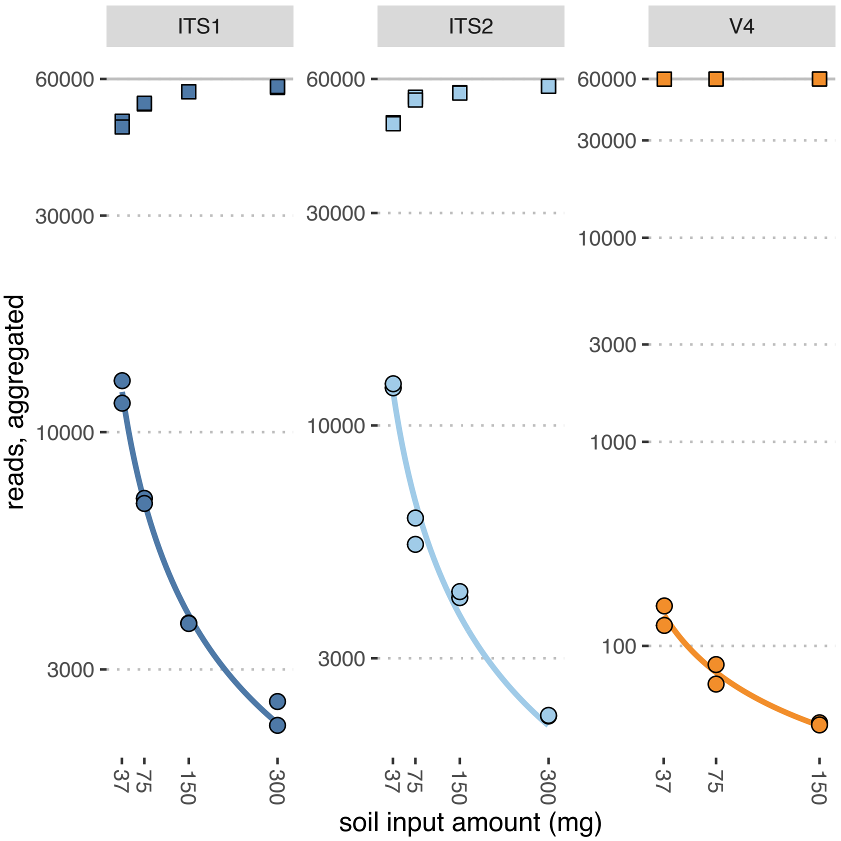

Fig. S3. Scatter plot of aggregated reads for the rDNA-mimics (circles) and soil-derived ASVs (squares) plotted against soil input amount (absolute amount of biomass in mg), for samples in which a fixed amount of rDNA-mimics was added to varying amounts of soil before DNA extraction. Each facet shows a different primer set and symbols represent individual data points. Count tables were randomly subsampled to 60,000 reads as indicated by the horizontal grey line. Non-linear regression (rectangular hyperbola) of the soil reads to the soil input amount was performed and used to calculate the rDNA-mimic reads shown as colored solid lines.

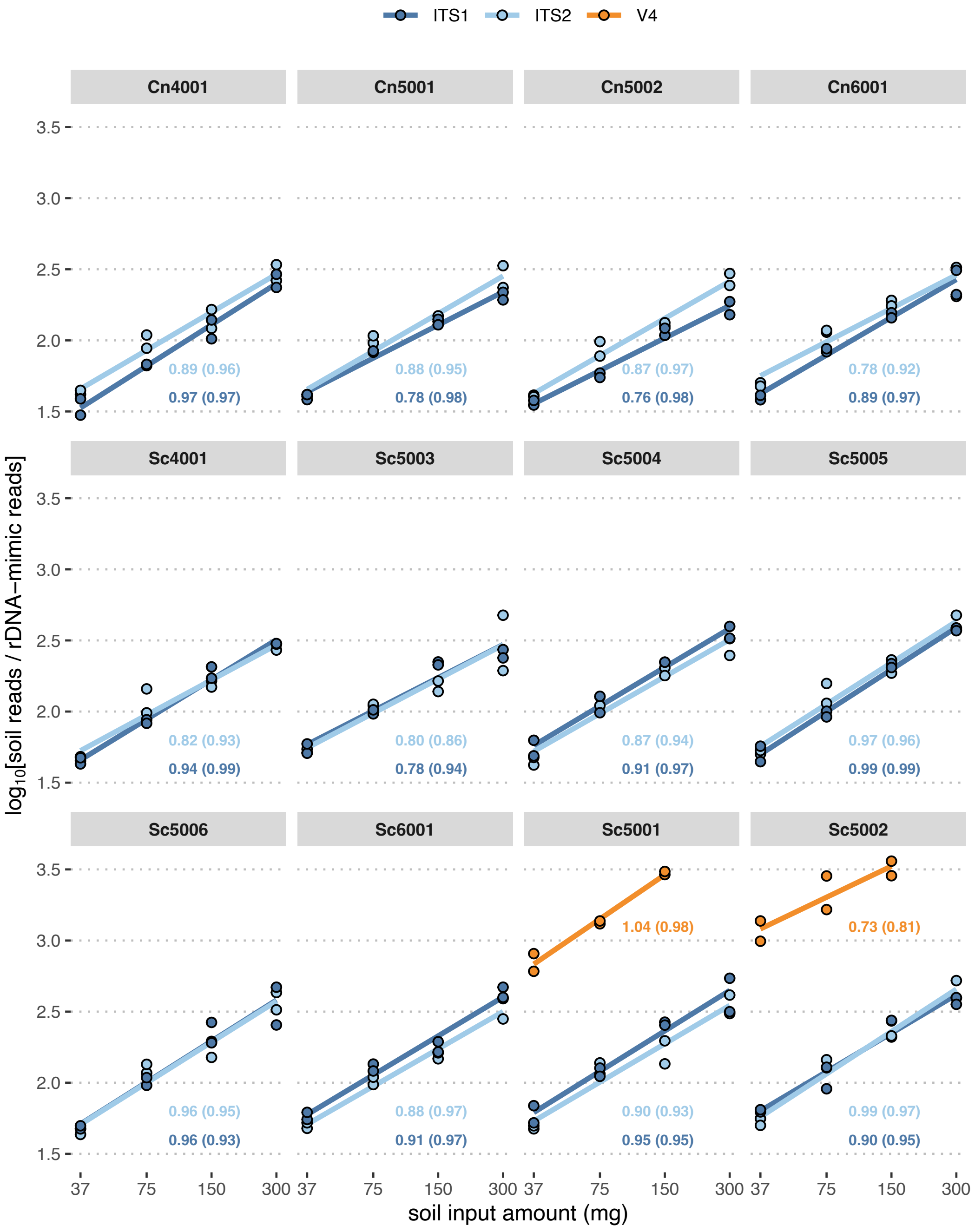

Fig. S4. Scatter plot of ALR-transformed soil read counts (*y*-axis) plotted against soil input amount (*x*-axis) for samples in which a fixed amount of rDNA-mimics was added to varying amounts of soil before DNA extraction. Facet labels indicate the rDNA-mimic set as the basis for ALR transformation. Symbols represent individual data points, colored by primer set as shown in the legend. Solid lines show the best-fitting linear regression models, on a log-log scale, with the corresponding slopes and coefficients of determination (within parentheses) shown as text labels.

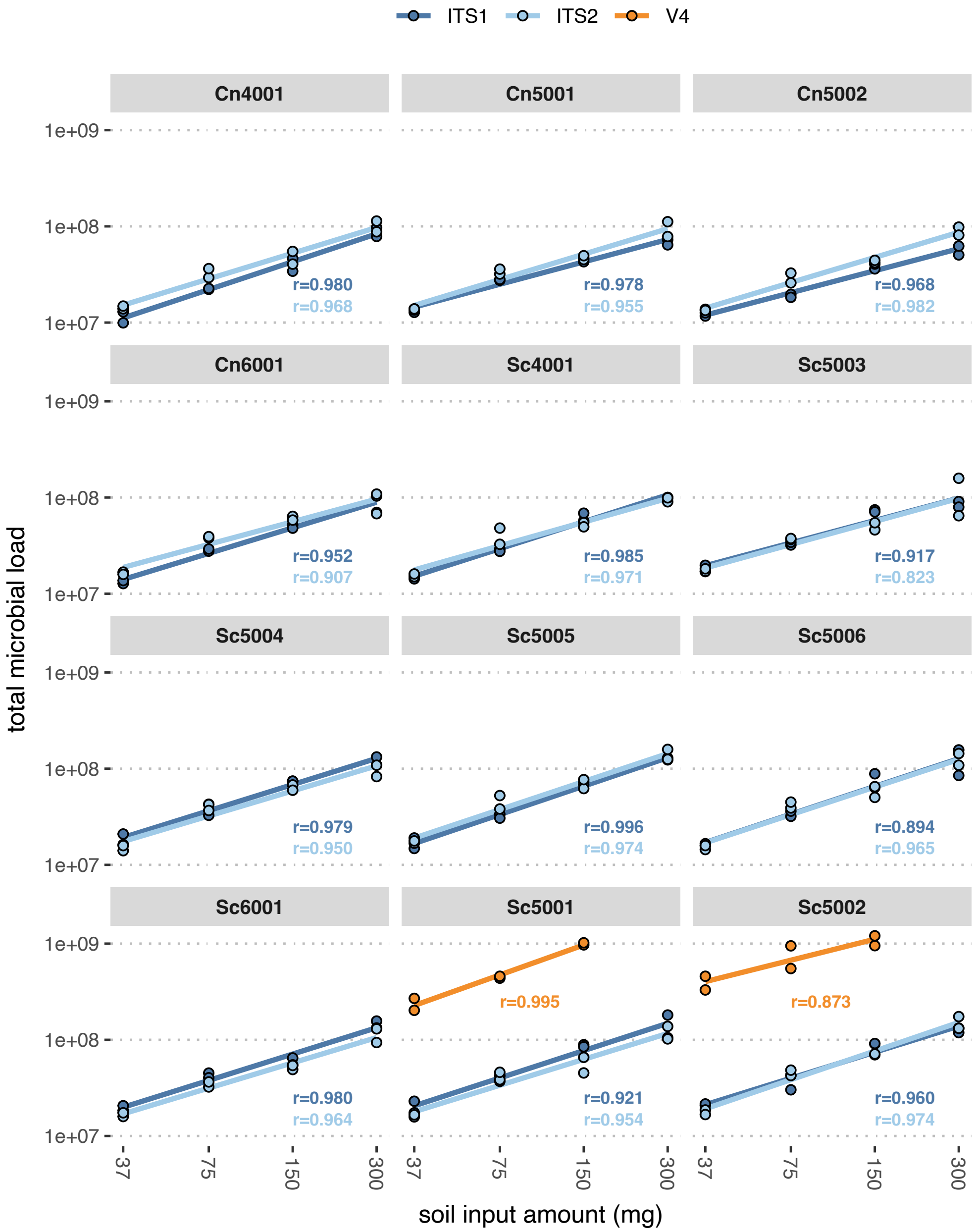

Fig. S5. Scatter plots showing the correlation between estimated microbial load (total copies, *y*-axis) and soil input amount (*x*-axis). Facet labels indicate the rDNA-mimic set as the basis for absolute quantification. Symbols represent individual data points, colored by primer set as indicated in the legend. Solid lines represent the best-fitting linear regression models, on a log-log scale. Text labels show Pearson's correlation coefficient, calculated on a linear-linear scale.

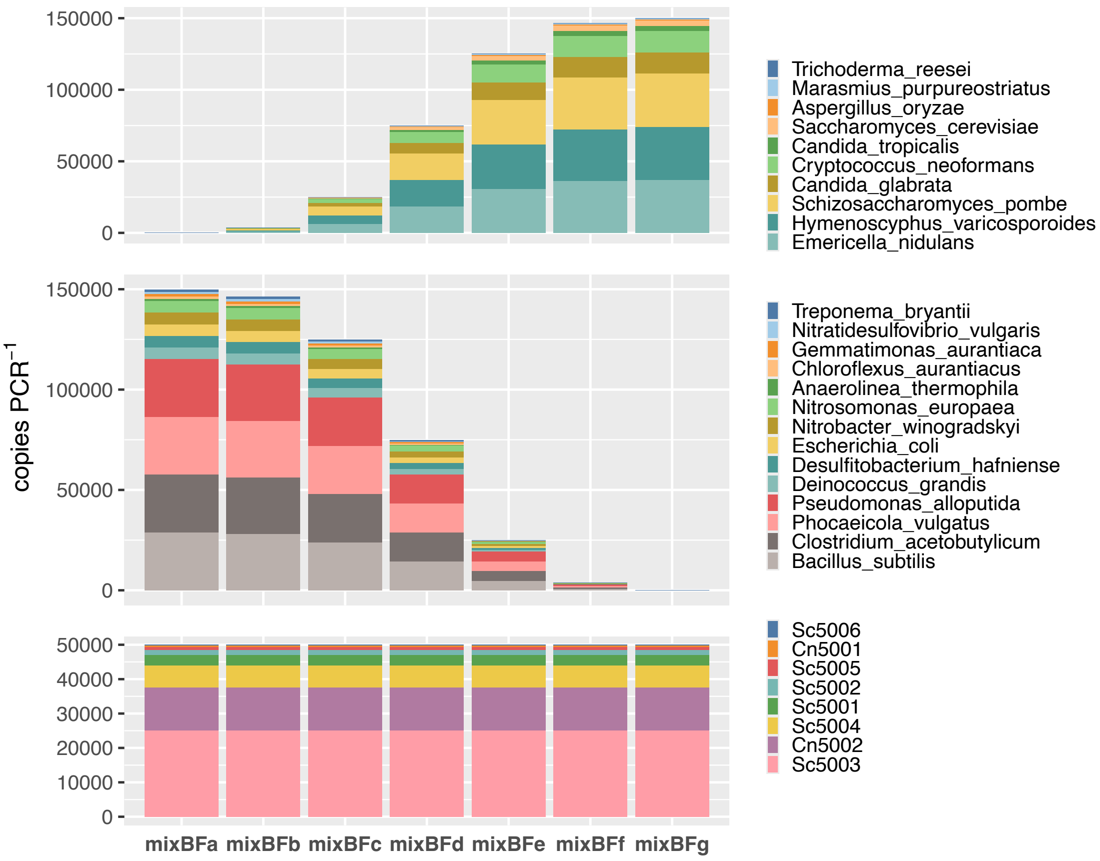

Fig. S6. Stacked bar charts of the compositions of mixtures mixBFa through mixBFg (*i.e.*, mixtures with varying bacterial and fungal loads and a fixed amount of rDNA-mimics). From bottom to top, panels show the copy numbers (per PCR) for the rDNA-mimics, bacterial strains, and fungal strains in each of the mixtures. Across mixtures mixBFb and mixBFf, the ratio of bacteria to fungi ranges from 25 to 0.04. Note that mixBFa and mixBFg contain only bacterial and fungal strains, respectively, and their BF ratio is thus undefined.

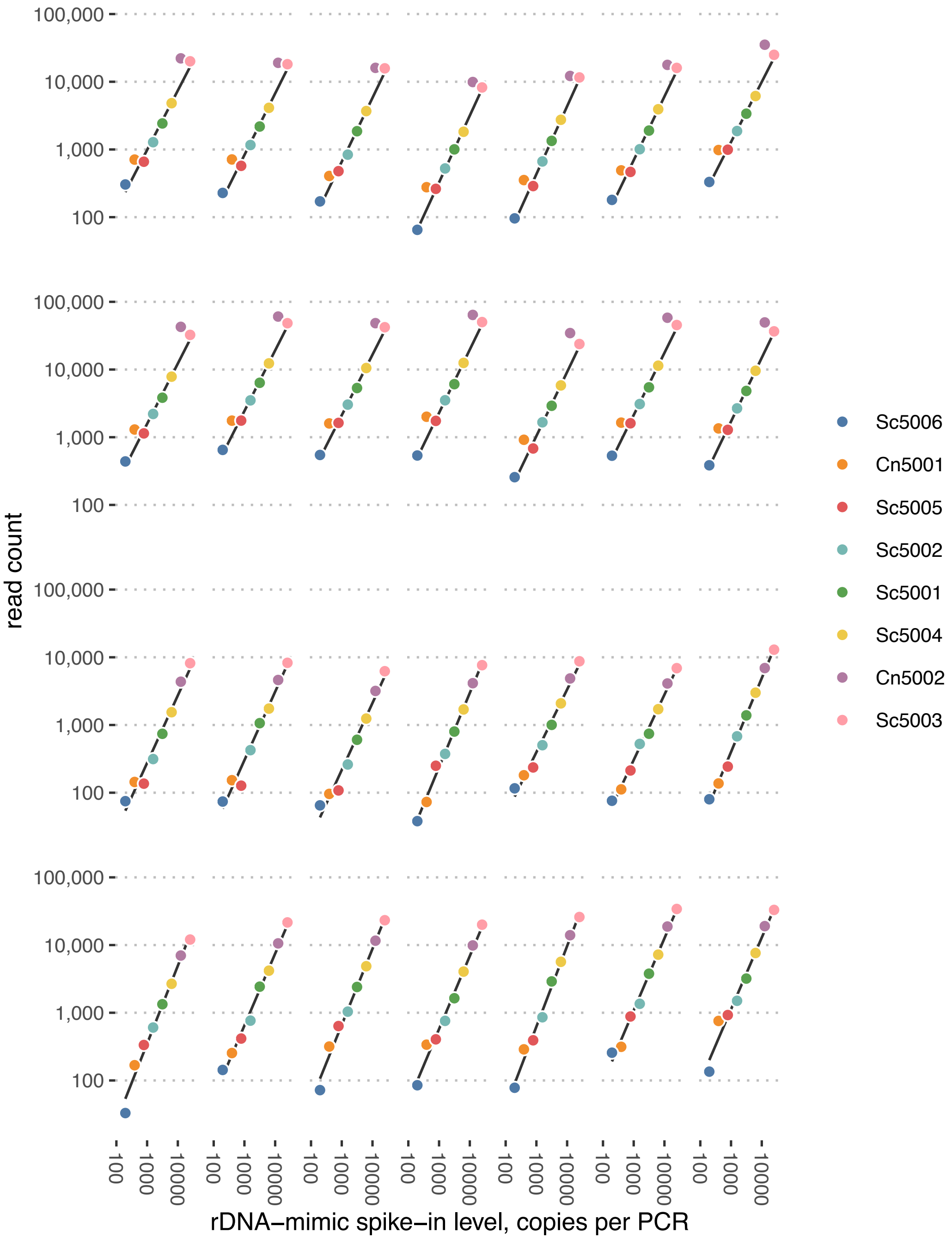

Fig. S7. Standard curves for individual samples. Each facet shows the results of an individual sample / sequencing library, with the top and bottom two rows showing results for primer sets ITS1 and ITS2, respectively. Each symbol shows a different rDNA-mimic as indicated in the legend. Solid lines represent the best-fitting linear regression curves, calculated on a log-log scale; note that only Sc-based rDNA-mimics were considered for linear regression for primer set ITS1.

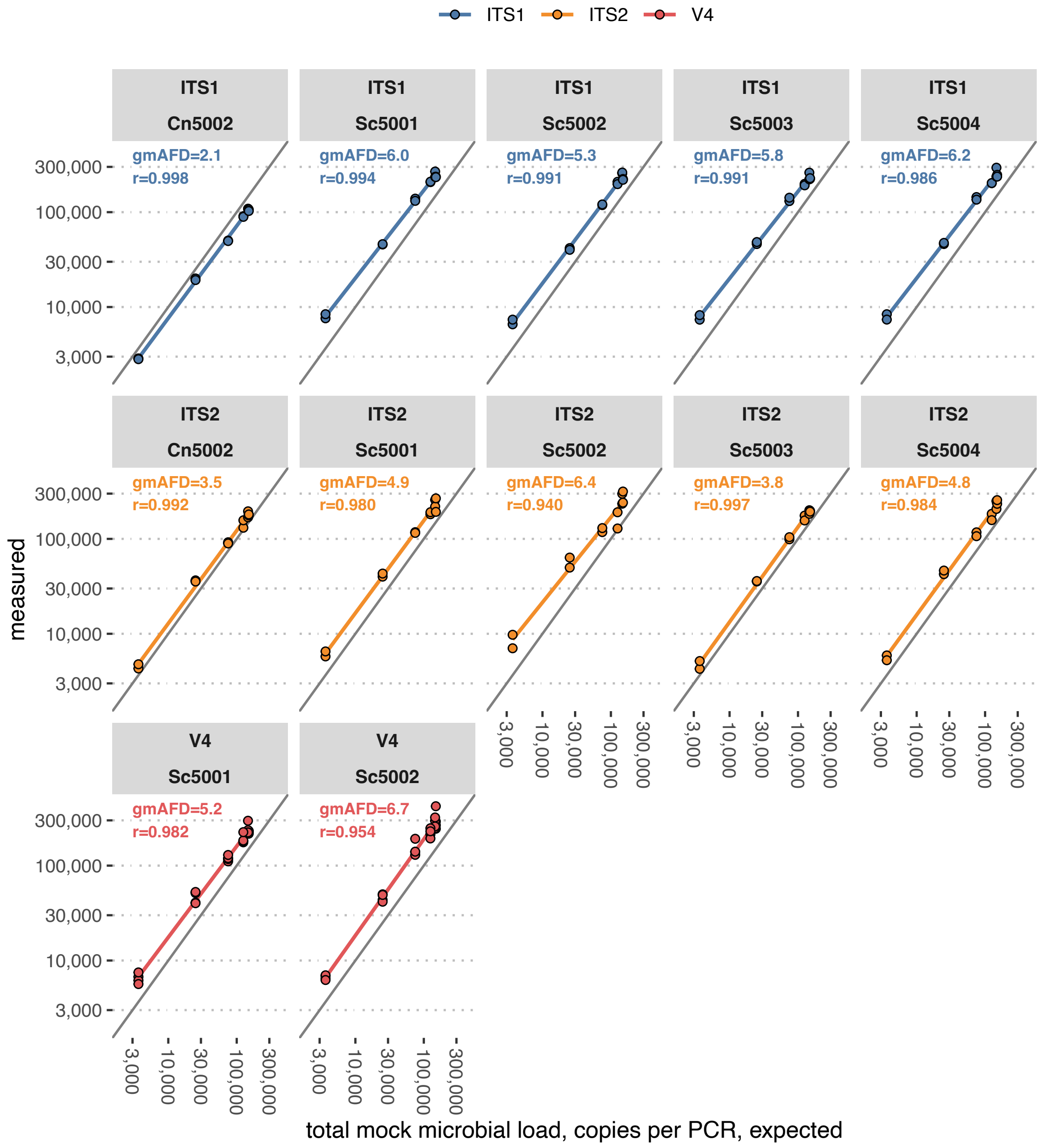

Fig. S8. Scatter plots showing the concordance between expected (*x*-axis) and measured (*y*-axis) microbial loads (that is, total fungal or bacterial absolute abundances) for the mixtures shown in Fig. S6. Each symbol represents an individual data point. Data are facetted according to the primer set and rDNA-mimic set as the basis for absolute quantification. The grey solid line shows the 1:1 diagonal, indicating perfect agreement between expected and measured values. Colored solid lines depict the best-fitting linear regression models, calculated on a log-log scale. Text labels show the absolute fold differences (geometric mean across data points, gmAFD) between expected and measured microbial loads, and Pearson's correlation coefficients (r) calculated on a linear-linear scale.

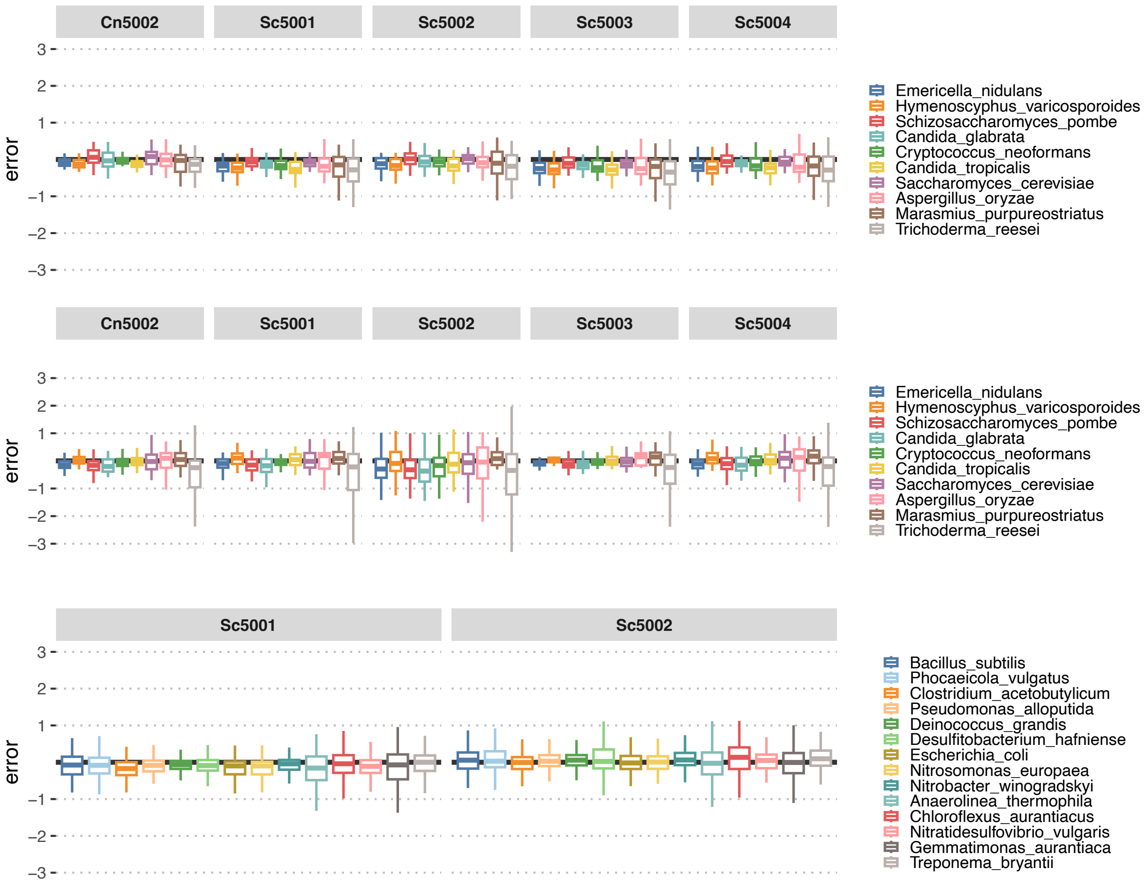

Fig. S9. Boxplots showing the (dis)concordance between expected and measured fold-differences ($\log_2$-fold differences) in species-wise absolute abundances, calculated for all possible pairs of samples. The top, middle, and bottom panels show the data for the fungal primer sets ITS1, ITS2, and the bacterial primer set V4, respectively. Each boxplot (outliers not shown) represents a different species in the fungal or bacterial mock communities, as indicated in the legend, sorted along the *x*-axis by decreasing abundance in the mixtures shown in Fig. S6. Facet labels show the rDNA-mimic set as the basis for absolute quantification. Note that abudance estimates involving bacterial or fungal strains with less than 10 reads were ommitted. The error shown on the *y*-axis represents the differences between expected and measured $\log_2$-fold differences in absolute species abudances between all possible pairs of samples.

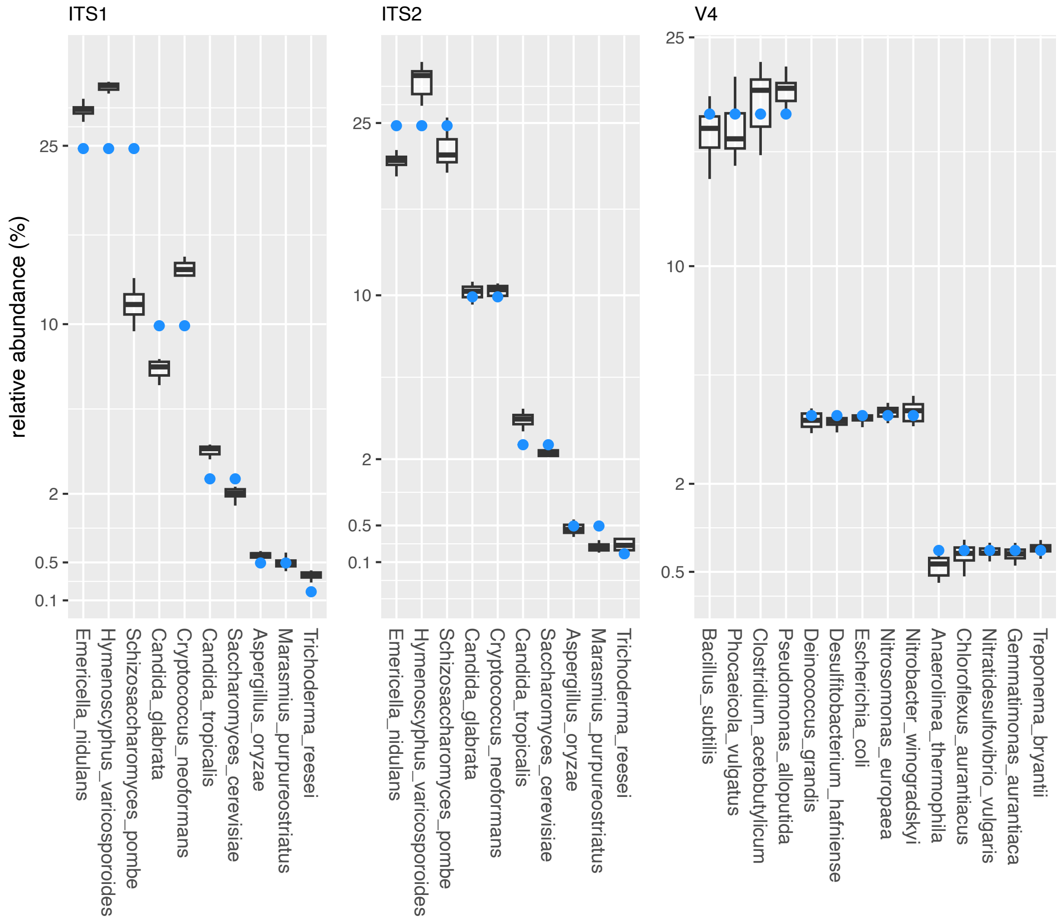

Fig. S10. Plots showing the (dis)agreement between expected and measured relative abundances of the fungal and bacterial strains for the mixtures shown in Fig. S6. For each species in the mixtures, boxplots (outliers not shown) represent the distribution of measured relative abundances across mixtures and replicates. Blue circles indicate the expected relative abundance of each strain, noting that these are fixed across mixtures.

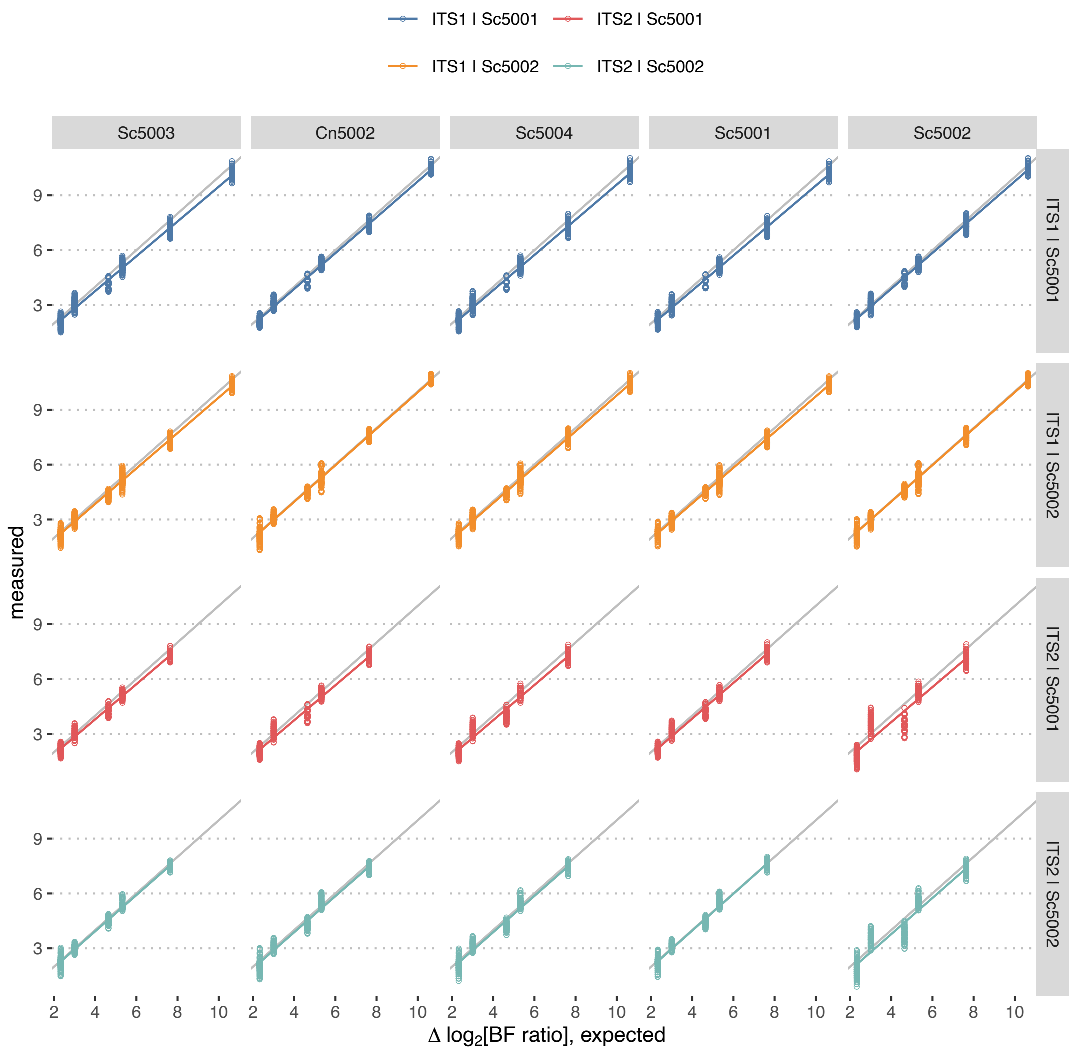

Fig. S11. Scatter plots showing the concordance between measured and expected differences in bacteria-to-fungal (BF) ratios for all pairs of samples/mixtures shown in Fig. S6, excluding mixtures lacking fungi or bacteria. Facet labels and symbol/line colors reflect the rDNA-mimics used as the basis for calculating fungal and bacterial loads, respectively. Each symbol shows an individual data point. The black solid line shows the 1:1 diagonal, indicating perfect agreement between expected and measured values.

Supplementary tables

Table S1. Overview of the rDNA-mimics.

Identifier	Genbank accession number	Natural rRNA gene sequence scaffold	rRNA operon regions	Length (bp)	GC content (%), artificial regions
Cn4001	PQ159722	*C. neoformans*	SSU-V9, ITS1, ITS2, LSU-D1D2	1320	40
Cn5001	PQ159729	*C. neoformans*	SSU-V9, ITS1, ITS2, LSU-D1D2	1320	50
Cn5002	PQ159730	*C. neoformans*	SSU-V9, ITS1, ITS2, LSU-D1D2	1320	50
Cn6001	PQ159732	*C. neoformans*	SSU-V9, ITS1, ITS2, LSU-D1D2	1320	60
Sc4001	PQ159721	*S. cerevisiae*	SSU-V9, ITS1, ITS2, LSU-D1D2	1596	40
Sc5001	PQ159723	*S. cerevisiae*	SSU-V9, ITS1, ITS2, LSU-D1D2, SSU-V4 (bacteria)	1919	50
Sc5002	PQ159724	*S. cerevisiae*	SSU-V9, ITS1, ITS2, LSU-D1D2, SSU-V4 (bacteria)	1919	50
Sc5003	PQ159725	*S. cerevisiae*	SSU-V9, ITS1, ITS2, LSU-D1D2	1596	50
Sc5004	PQ159726	*S. cerevisiae*	SSU-V9, ITS1, ITS2, LSU-D1D2	1596	50
Sc5005	PQ159727	*S. cerevisiae*	SSU-V9, ITS1, ITS2, LSU-D1D2	1596	50
Sc5006	PQ159728	*S. cerevisiae*	SSU-V9, ITS1, ITS2, LSU-D1D2	1596	50
Sc6001	PQ159731	*S. cerevisiae*	SSU-V9, ITS1, ITS2, LSU-D1D2	1596	60

Table S2. Sequences of the rDNA-mimics. Artificial sequence regions are shown in lowercase.

**Sc4001**
TGATTACGTCCCTGCCCTTTGTACACACCGCCCGTCGCTAattgtcagtctagcgaatcattataccgaagaacatccgtttatgagaacgtgctaccaattaactgtactaagctgtccAAACTTGGTCATTTAGAGGA<br>ACTAAAAGTCGTAACAAGGTTTCCGTAGGTGAACCTGCGGAAGGATCATTAtcataagcagagcctttatcccatataagctattgtcacgaagtgtcactgtgaacgaatgttctctaaacttactacggcttcagatg<br>taacggattcagactactctattcataacggactacagattgcgtcaactacgatattctcttgagatcacgattagcaagtacctttgcagcttgaaattaaccagacctttccttggaatgcctatacagagatttat<br>cataccaggagttctccagattacctagatgtcttaacgagatacaggacttacacgatgacttagtgtgttgtttgcatcaacctaacagtaactgagcgaattgtaccaacgtattctttaccggaagtAAACTTTCA<br>ACAACGGATCTCTTGGTTCTCGCATCGATGAAGAACGCAGCGAAATGCGATACGTAATGTGAATTGCAGAATTCCGTGAATCATCGAATCTTTGAACGCACATTGCGCCCCTTGGTATTCCAGGGGGCATGCCTGTTTGA<br>GCGTCATTTagttgtctgccagaaatcattgaacattccgacgaatatcgacatggttgcttatctaagaccttaaacggtacttggttagctgatcgcaatacttgaaagacttgatcctgtacttacctggacacgat<br>gtaataatctcacacagttatgagaagctggttgcacctaaatagtcaattagcacgtagtaacgtagacttgccactgatgaaacataGTTTGACCTCAAATCAGGTAGGAGTACCCGCTGAACTTAAGCATATCAATA<br>AGCGGAGGAAAAGAAACCAACgaacgattgaagatgtactcagatattcattgatgggcctacgtctacttactatgggaatgtaaatactctgttccagcctaaggttagctttgcgaatacaaatgttcttatcgacg<br>cacagtcatacggattacgatcaagttaatggttactccctaccgattattgcatccagatcatattgagaggaatcacctgtacggtttagaaatcagctctactagaagacactattgccatacgtcaaattgcagtg<br>agtttcaccaaatcatggagatgttacccagttagcatacaactctttgcacaagtgcataatgtagtccctatgtcacaaggttatacgaagcatgtcaaatcatcgcctttagttacgatgtagttccacaagcgaaa<br>ttagtttccgaaatggtcaagcatccaagtttagctcgaatctttaaggagatactcgaagtgcctatattacggaggtattatcatgtagcaagcgttacctagcttattagtccacgaatcatgtgttagaagtcgtc<br>aagttcatgttatcctaccagCCGCCCGTCTTGAAACACGGACCAAGGAGTCTAAC
**Cn4001**
TGATTACGTCCCTGCCCTTTGTACACACCGCCCGTCGCTAttactgatcgaacgtcgtataatgctgaggcatctgttattaaccgtacctttcaaggattaccatgtggcaacataagtAAACTTGGTCATTTAGAGGA<br>AGTAAAAGTCGTAACAAGGTTTCCGTAGGTGAACCTGCGGAAGGATCAcatccttggtctaagaaagtgcatgatttgagcataccaatcgccattacgataaagatcctttgagtctaacgtacactgtgtcatctgta<br>agataccattgtcactacttcagtcagaACTTTCAACAACGGATCTCTTGGCTTCCACATCGATGAAGAACGCAGCGAAATGCGATAAGTAATGTGAATTGCAGAATTCAGTGAATCATCGAATCTTTGAACGCAACTTG<br>CGCCCTTTGGTATTCCGAAGGGCATGCCTGTTTGAGAGcattgaacacttcgtaaggtacacctatggatcaacgattaagtctcgataccgtaagatggtaactctagtcagtgataatcaacagcgtagtacattcgt<br>aagcagtcttggacattactttctgagtgcaacattcaacgtctaaacgggttaaatctctcataacggaacttgtgtgcaacagatgctatatggtatgcaaatgcgatacactttgACCCGCTGAACTTAAGCATATC<br>AATAAGCGGAGGAAAAGAAACTAACgtaaagctattaaccggagtgaatccttcattaaagtcgcacaagctgtattaccgttacgcaacgtatttgattgaccatgtgaacagaagtaccctattgacctagattatgc<br>agcaatgcctaagactatttgcctaattcgggctatttagaccaatcctccatgatgtatatcagtcaaggctagtttggaacatacacgaaagtccttatgtagtagagtgcaattctcgtatccttcaacagtgttat<br>cgagtatcgaacgattatcctatgggtatccacttatagaacgtgtgtagactaacctgtaaacgatgtctctgaaagcaagactacttatctgagatcggatgtttaagacgctatgacaccattaacttatgccagtg<br>ctagtcattatgaccacgatttggaatttatggctatcgccactatgaaatgctaagctacctgaacaatttgtacgcagtgacagtagatcctttgatccagaacttattaagagctgaccctatgaaacgtgatgtcc<br>tattcattattacgggaaaccgtagCGACCCGTCTTGAAACACGGACCAAGGAGTCTAAC
**Sc5001**
TGATTACGTCCCTGCCCTTTGTACACACCGCCCGTCGCTAttggccttcagtcgagaacttgttgaaactgtcctgacgcactggaacgagcttccattgattcgctagaaatgccgaccAAACTTGGTCATTTAGAGGA<br>ACTAAAAGTCGTAACAAGGTTTCCGTAGGTGAACCTGCGGAAGGATCATTAcacagtgtggatctgacgaattaccaaggcactccatgtgtgccatctacgtctcaggaattgtacctgctaccactaggcatcgagaa<br>cgctgcatgtattcaccgagtaaggtcttccagactccgataccgtatgtgttcccaggagaaatgtcgcttagccggttcaagccatcatgtgctagactagacacgtctatcgcggtttacacgaccatcagttgagc<br>caatgctatccttgcgggtcaaacagagcttacggatcacccatagttgtcacgccacgttaaagttccgagcgaaacgctatctcttcgagagctgtcccaatgaaactctgcacggacttgtattgcacAAACTTTCA<br>ACAACGGATCTCTTGGTTCTCGCATCGATGAAGAACGCAGCGAAATGCGATACGTAATGTGAATTGCAGAATTCCGTGAATCATCGAATCTTTGAACGCACATTGCGCCCCTTGGTATTCCAGGGGGCATGCCTGTTTGA<br>GCGTCATTTactatgaggcccacagttacgaacgactagaccactgtcttacgagtgtcgcaccataagatggcgagtaatccgctcaatccactggttcctgagaaagagccggaaatctgaggtcattctgcccatga<br>tagctggaaacacccgagtctctaagtgtgagtagcctgatctactgcaaacgcccgatacatatcgtgagagtctgctaggactgatcGTTTGACCTCAAATCAGGTAGGAGTACCCGCTGAACTTAAGCATATCAATA<br>AGCGGAGGAAAAGAAACCAACtcaggctatattgaggcaccgcctggctagtagattacgacagctataacttcgggcaagccggttgatccaactatcgaaacctcgttagagcagtgtgtggcctaatggcatactgg<br>aacctatctgttacgccgagaactcgtgagcaactcagtctcataaagtcatggtccgcactgatgctgcacaaagctaccgattgatacgttcgccgactgtgatgcgtgaatcattccgtcaaagtgtccacccgtgt<br>aggcattggtatatcgaccgatccaagaagcgacgcttagtacgcgattacattgggcagatggtacagctcccataaacgctaggaactgttcgcaagagtcctgtgtcagagtcaaggataccgttcagaggcaaact<br>gaccgtcattcgtgctaaacgatgtgatccgccctttcagacgctagtgttacctggaagaagattggcgctacctatgtcccatacagcgacaaggtcttgtagaaggcatgtcaagctccctaaatggctccgctaaa<br>gtacgtgttgagggtctccaaCCGCCCGTCTTGAAACACGGACCAAGGAGTCTAACaaaCACCGGCTAACTCCGTGCCAGCAGCCGCGGTAATACGGAGGGTGCAAGCGTTAATCGGAATTACTGGGCGTAAAGCGCACG<br>CAGGCGGTTataagagctttgagcccacccgcatactgatttgactgccttaacttggtgaagccctcggacggaaacttgacatctcgttctatctgaatgagcgcggcacagcttgagtctacttggaattgcattag<br>caccggcctgccttacaacactgttgcgtattggactaactagcggcctGTGGGGAGCAAACAGGATTAGATACCCTGGTAGTCCACGCCGTAAACGAT
**Sc5002**
TGATTACGTCCCTGCCCTTTGTACACACCGCCCGTCGCTAcctagaaagctcgccattagccgcagtagtgattggacatcagagtttcgctcacaacgtcaccgctcgttatggaacttAAACTTGGTCATTTAGAGGA<br>ACTAAAAGTCGTAACAAGGTTTCCGTAGGTGAACCTGCGGAAGGATCATTAaagcgttggttcgttacgcaaggctctacgaaagcagtgtctacttagcgttcagtgcagcgatccacaatctcatgggtatgtcatcg<br>accagctacgacgcaagtttcccagatcaagattaggtgcccttcaagcacggttggaactctaccgacaattacgaggtcccaattacgggtggcaactatgctgtaccagtaagatcctgccgattcgacgcacagtc<br>ataactcagtgtacgtgtatcctggcaaggaggaagctccctttacatgctagtgcaatgtccgcagtttgcgagaggactatatccagtctaccacaggtcagaggttacaccctggctatctagtatggAAACTTTCA<br>ACAACGGATCTCTTGGTTCTCGCATCGATGAAGAACGCAGCGAAATGCGATACGTAATGTGAATTGCAGAATTCCGTGAATCATCGAATCTTTGAACGCACATTGCGCCCCTTGGTATTCCAGGGGGCATGCCTGTTTGA<br>GCGTCATTTaccgtaaagctaggtcaggtcttcactgggcaacgacataatgggtaactcacttccagcctacatcagcggtgtcaaaggtagatgcctatcgtaccacccacaatgctctagggtttcagagaagctgt<br>gtcttccgatggtcaccagatggattcgactcaaggtcatacaggagtgtcgcgtaacatagcctatgcaaccgttcggttaaggacgtGTTTGACCTCAAATCAGGTAGGAGTACCCGCTGAACTTAAGCATATCAATA<br>AGCGGAGGAAAAGAAACCAACgctgcttagcctataccgtaatcggtgtgcgtgaacactagccaggtactgaatctaggatcgctgtggatctaaccagtccgctacgacaagagtttactaggaccgcctaaatcatc<br>ggcgcttaccgttaagaaacctgtccggcgacatatacagtgccattgcgcttgagaatcatgctgtgcgagagacatacacggttccgagttgacatctacgtgaagggcatctttcgatgctgacccgaagtttatct<br>gggaagctacgtcatttgcctaccgctgcgactaatctttgcagacgacatgctatgagcttgctggaccacgaatcgttaccagtcatctgagacacttggcatacgcttgggcttgatacacctatggatgggataca<br>ctgatcggctgccgcataatttgctacgccttacagagaagtgcagtctaccggctgttaatactccggctttacacgagaagctactgagggccatttgacacaatcgcgtgagtttgctgatctgacatgggctgaaa<br>catgagcctccgaactatcgtCCGCCCGTCTTGAAACACGGACCAAGGAGTCTAACaaaCACCGGCTAACTCCGTGCCAGCAGCCGCGGTAATACGGAGGGTGCAAGCGTTAATCGGAATTACTGGGCGTAAAGCGCACG<br>CAGGCGGTTgtagttaggcaactctaggcggcaactgctcatcaactaggagtacagtcaatctgacggacgcgctactgcatacttagtcatctactggttccagagccacgggtcatcgtaaattgggtattccgaaa<br>tggcccacacgccgttcacgtttcaaatgattggcatctagggacacctGTGGGGAGCAAACAGGATTAGATACCCTGGTAGTCCACGCCGTAAACGAT
**Sc5003**
TGATTACGTCCCTGCCCTTTGTACACACCGCCCGTCGCTAtcaggaagtgtgtcccattgccggaggagtcctattgaatcacggattacgtctgtaacgctggaccgaggttgtatcatAAACTTGGTCATTTAGAGGA<br>ACTAAAAGTCGTAACAAGGTTTCCGTAGGTGAACCTGCGGAAGGATCATTAgcttcgattacgatgcccaaatacgatccgcgtagtttccacgaggtctacagtaccctattgttcgaggcagtaacctgaaccgcgtc<br>tgtcaacagttatgtgacggcaagttgtccaagtccgagccatactatcagtcgtcttagctcatgggaagctcgcagtgttaagctcagtaggcaaattccagcgtgatgccgatccagtgtacgagaatccttacatg<br>caagtgtcgcaggccagatcagtttcgagaaagagtacgttctatccctggcgtcctcagtgactcaagatgagattacatccacacggtctcggtccattcgcaaagtacagtgtttccttagcagcaggAAACTTTCA<br>ACAACGGATCTCTTGGTTCTCGCATCGATGAAGAACGCAGCGAAATGCGATACGTAATGTGAATTGCAGAATTCCGTGAATCATCGAATCTTTGAACGCACATTGCGCCCCTTGGTATTCCAGGGGGCATGCCTGTTTGA<br>GCGTCATTTaacatgctgcgtagtacgtcgatcaccaagctatgagcgttgtcaaaggagtgtcaaccgacgagtccaggtttcatcaccttgctaggtatccacaggtgcattaggcggctaagtcttccacatcgtat<br>tgccgaagtgtatcgcccagacattcaagctgtcagaactctgcgttacagaacgtgccgtcaagattcaggctatcatccgtgaaccaGTTTGACCTCAAATCAGGTAGGAGTACCCGCTGAACTTAAGCATATCAATA<br>AGCGGAGGAAAAGAAACCAACtacgtgagatcggtccgatatgagctgtccacaatagccatagactaggagtcacccttcgagtggttctagcacatccagatgacacactaagtgccctgttcgggacttgtaaagca<br>cgattccttggttaagacgcctcccagtcagtatcatggtcgtaaagttcgtccagtggtcaacgctcttcgtcaagcgataagttaaagccggtagctgctcaagcctgccatacggattagttcaaacgagcctgtcg<br>tgtacgttctccgcacaatgtctaacaatggtacggtgcagatagcttccgcccaggttattaaggcaaattggcccatccattctgtcggtcggcaaacagttcctgaaattccgctgaggttgtaagacccggtctga<br>atagccagatcaatacgtcggtgctgatgagtgccatcacagtttctctaggatagcgcacgttcatgtcgcgtaacgcatctagcatttaggtgcaacggtactacgtccaccagtaggaagttcgcataaacggtcac<br>cttagcctgagtagccgtcaaCCGCCCGTCTTGAAACACGGACCAAGGAGTCTAAC
**Sc5004**
TGATTACGTCCCTGCCCTTTGTACACACCGCCCGTCGCTAtcccgcaaatacctttggagtgcgtcactatctaggagtgtgccgatgactcgtaatctccatcctcgaagttgcacgatAAACTTGGTCATTTAGAGGA<br>ACTAAAAGTCGTAACAAGGTTTCCGTAGGTGAACCTGCGGAAGGATCATTAataatccagggtccacgagtgaatgccctgcaaatgtaccaagttcctgaccttctggcatgtgaagccgatcttatcgctgaagagtc<br>tcgaagtcgctgacatacacccgtattgtcgatctgttggcgtaacggacatacgatgcactgacagcagttgcttagagcctagacacgacattgccttgaacgaccttgctactcatagggatacccgacgtagacgt<br>ttagtcctgcaagtcgaaagccctttgtgagagtcgccttatagtaccggatagtctcccagccatattggagagtccatatagccacggtagaatgctccgaggtaacctgagtcaaattgccgcactagAAACTTTCA<br>ACAACGGATCTCTTGGTTCTCGCATCGATGAAGAACGCAGCGAAATGCGATACGTAATGTGAATTGCAGAATTCCGTGAATCATCGAATCTTTGAACGCACATTGCGCCCCTTGGTATTCCAGGGGGCATGCCTGTTTGA<br>GCGTCATTTagtgacagttcacggtagcagctaaatcttcgggcatcacgagtacatgagtctcccatcgttaatccagcaagccgatgtggagctatttcaacgggacgtatatgtcgtccatccgagttgcggactat<br>ctacagggtgaattatgcgactgactgccttgccactacgaaacagtgcgttcaaattgcgctaagggcgtgcgaatacttatgcaggcGTTTGACCTCAAATCAGGTAGGAGTACCCGCTGAACTTAAGCATATCAATA<br>AGCGGAGGAAAAGAAACCAACatgtccaaccgaaactcgtgatcttagtgaccgcacggatctgtcattcgagaagcgtagagacttatgcctggccttaacttgtgctcagtagcctcaagagaactgcctcctgtct<br>attacgggtaaactcctggtgatccagagacgtagtgtcagaacagcctagatgtgttgccacgacctgtaaacggctttcttacgacgcaatgctgatggtgactggcgattaacgaaccgaatcatcctgtgtgcatc<br>ctacggtgtgccatttgaaccagagagtatcttcgaccacgatctgcaagggtgtcatgcttgacctagagtaccacgttcagttgcctcatagggcttagcagcgtattcatgcgacttgcgataacgatgtcctgtac<br>ggacgttccatagtccgacaaacccatgtatgtctgcgagaggttagccaagagtgcttactccacctagtgagatgtagcgacaacgactgtgagtgtacgactccttagggtatagcgttgccaaacttcccaaggta<br>gggagcctttcccattacgaaCCGCCCGTCTTGAAACACGGACCAAGGAGTCTAAC
**Sc5005**
TGATTACGTCCCTGCCCTTTGTACACACCGCCCGTCGCTAgacaccctgttcagattagcgagcctcagttacaccagattccgagttcgtaagatcgagaggagccatcatggacgtttAAACTTGGTCATTTAGAGGA<br>ACTAAAAGTCGTAACAAGGTTTCCGTAGGTGAACCTGCGGAAGGATCATTActgacggaccaatctgtatgtaaagcggctattcaggagcctatccgacgagttgatgcttacaaggcgatctatccctgaccagtgct<br>aaccatgtgcataagagcagtctcactcacgagtctcggttccttagacgattcaatgccaagttgtgccggagaacacctgttgatcctcgacaatgattcagtccaccgggatgtctgtagttcccaacgccaatatg<br>tagagcttcggtccacgaaagtaccgtggtagccatgatatgacttacgcccgacaaagttcgggagtttctcgcatgtgaagtttccgcaaccatgagcaaggtcgtttgacctggaagtgtatgatccgAAACTTTCA<br>ACAACGGATCTCTTGGTTCTCGCATCGATGAAGAACGCAGCGAAATGCGATACGTAATGTGAATTGCAGAATTCCGTGAATCATCGAATCTTTGAACGCACATTGCGCCCCTTGGTATTCCAGGGGGCATGCCTGTTTGA<br>GCGTCATTTatctgacagccttctacgagcctgctgaatcagatgaaccacttggtcgcaatgatcgcaaggtcgggtatatcttcacggttagatccgaactgctccactgggtacaacacactgacttggtaactcgg<br>tcatacacgtcgggaacataactgcctgtgatagcacgcactcttaggacagtcgcattctctaggtcatggaatagcgcaacatcgctGTTTGACCTCAAATCAGGTAGGAGTACCCGCTGAACTTAAGCATATCAATA<br>AGCGGAGGAAAAGAAACCAACtccacagtatcatccgatggagcgattcgcatacgacagtcaatggctattggtcaggacctagcttccaagtcaagggaaggtttcaggatcgtcgcatcgtactttcctacgaagtg<br>cctaaagggatcactctccgaacggtttgtatcagcgtgcagatgtacctgttacgccagaggaatgacattctacccgagggatcttacagtccgggatttgtgcaatcacagttgggctctaacgtcaagcgaggtgt<br>atgtcccatgaataaggacggctttctcaggccaagaagtctacgcagaagttacccagctcgtttacggtgtccactcaaagtctagcatgttccggtgacctagttgatggcagtagcagtaccatgacaagaggctt

ccgattatccagacccagttgtgggctaatatgagcagcaccctagtatttcgcgcaatgccggttatatgaaggccacgtacaagtttctccgcgcatgtgtcagatagtatccggttccacagcataagtccgccagt<br>tggttcactaagttgccgacaCCGCCCGTCTTGAAACACGGACCAAGGAGTCTAAC
**Sc5006**
TGATTACGTCCCTGCCCTTTGTACACACCGCCCGTCGCTAcatgactggaaaccctctgacgtgtaactctggaagctcagttatcggaaacggcgctaagctacgtgatcgtaagcagtAAACTTGGTCATTTAGAGGA<br>ACTAAAAGTCGTAACAAGGTTTCCGTAGGTGAACCTGCGGAAGGATCATTActctgatggacctggtgatacacggtactatttggcatggtcacatcgggcatctgtaagacctccagttgtagtgtgcagagttccca<br>gacagtctaagacggcattgactatggccttgtggttcgagaaccgaacatccaagagtttcgctcgttcatggcgataacccttcaacgtgtggtaacctgtaacgcagtcagctttagcgcgtgaataccttgaggca<br>atacaccgagttgtgctaccctagtgatgacagaatggcaccttatgctccggtacacctacggaatcatgcaagtggaatccctttcgagagcaggctcagtttagttgcgaagtgatctccgcatttccAAACTTTCA<br>ACAACGGATCTCTTGGTTCTCGCATCGATGAAGAACGCAGCGAAATGCGATACGTAATGTGAATTGCAGAATTCCGTGAATCATCGAATCTTTGAACGCACATTGCGCCCCTTGGTATTCCAGGGGGCATGCCTGTTTGA<br>GCGTCATTTaacttagggagtatgccgtcgaacatcgctcgtgagtaacttatcgtgcggatacacctcgtacatgccactcggtacttagaatagctggtaacctccgatgctcgcaatgcgtagttctggattccaat<br>ggaccaacggtcattcctgggtgacaaagcaatctcctgtagcaggtcacagttctcgtctcgcagtaacgaagtcctcttacgtcatgGTTTGACCTCAAATCAGGTAGGAGTACCCGCTGAACTTAAGCATATCAATA<br>AGCGGAGGAAAAGAAACCAACtattgacgaccgttgccagagagccatcacttggtttcgactataacgacagatccgtggcctcctaaagttgcgtatgcagtatcgagatgtaccctgcgaaccgagtgtactaacgt<br>gtctgaggaatccattcccgtatcgggcacaacagtatgtgtcttccagatagagggcctttgctgacgaagtcctagactatcgcttagagacgcctacagaccagtaatcgtgaccttctacctgagatgccgtgaac<br>ataggtgctaatccgagagcatgtgtacgaactccgaaccttgccattaagggatgagcctactgaactaccgctgatcgtgcgagtatatcctgctgctaacgtaaactcctgagggctacagctaaacagcttggacc<br>tagtgtcatatcgccgttccaactgactccttgagagactgcgtaagatttccgccgacattgccaaacgctaattgccgatggtgtaaacgacccgcattccattggttgctaaagcctcgtaagaatccgggctgact<br>atcatgtgagcttgacgctacCCGCCCGTCTTGAAACACGGACCAAGGAGTCTAAC
**Cn5001**
TGATTACGTCCCTGCCCTTTGTACACACCGCCCGTCGCTAgcacctagcctttaacgagaagaatgtagccctacgccatcggcatgtgattccatacgatgttacgaaacctgaggcagAAACTTGGTCATTTAGAGGA<br>AGTAAAAGTCGTAACAAGGTTTCCGTAGGTGAACCTGCGGAAGGATCActtctgaaactatgacgcgccaaccggaatcgtgtaatggattgacctacttgctcggacgacggataacgctgtatgcaaatgtgcctgta<br>actcggctctgcgaactgctctgatctaACTTTCAACAACGGATCTCTTGGCTTCCACATCGATGAAGAACGCAGCGAAATGCGATAAGTAATGTGAATTGCAGAATTCAGTGAATCATCGAATCTTTGAACGCAACTTG<br>CGCCCTTTGGTATTCCGAAGGGCATGCCTGTTTGAGAGtccacgtaaatcagcgcgttatgggtctgacgtaagcacaagggtcctatacacgctactctggttatccctgagaagtcggttaccatgtcacacagtcag<br>gctatatgccctcacgttgattcgagcgaagttactgcaccaagtctggcgtagttagtgttccgtagagcaagtcactcaatcccgagcaaagtgtcgtgatgctgttcagcaagacACCCGCTGAACTTAAGCATATC<br>AATAAGCGGAGGAAAAGAAACTAACaggtcctcagaggctaatgtttcatgcaatgagatcccgcgtggacaccaccaagattctactgttgtcaagatacgggcgactcgacatggagctactattctatcagaagagc<br>cctgccaggcgttcaatcgcatttccatttaatggctgactcgcgcagacgaagtctcctagagttaagtcttacgagcaccgcttgtgtgagcacgatcatacgatactgactaaggcgtcaccgagtttcagacccta<br>cgacatgactgtctttaggccagagtctactagaccgagctttggatgccaacctttccgaagtgagatttacccacagcgttcgtgtgttcgactaacccgcaaagtgttaccataggctggtcctatttcgcagtggc<br>tagagagcaatgttccaggatgtgctactacttgccgtgagctagacataccgatggctaagtggatacgttacaggcgcacgtagttctaaccggcttatacggataacctgacccgagcgttattcttatgccgcaga<br>gaggtttcttacccgaaggcactagCGACCCGTCTTGAAACACGGACCAAGGAGTCTAAC
**Cn5002**
TGATTACGTCCCTGCCCTTTGTACACACCGCCCGTCGCTAtgcggagcatcctagtacaatatccggttgcctataagcccggtatgcgcgaattaacctaactgccagagatgagttccAAACTTGGTCATTTAGAGGA<br>AGTAAAAGTCGTAACAAGGTTTCCGTAGGTGAACCTGCGGAAGGATCAtaggtcacgctagtaccaaggagactcagaccttacagcttgcttgcagacagatcggaatcccacagcagagtttagacgtttggagacag<br>tcccacttcagtcgttggatgcacttagACTTTCAACAACGGATCTCTTGGCTTCCACATCGATGAAGAACGCAGCGAAATGCGATAAGTAATGTGAATTGCAGAATTCAGTGAATCATCGAATCTTTGAACGCAACTTG<br>CGCCCTTTGGTATTCCGAAGGGCATGCCTGTTTGAGAGcagggttccctagtaagtacgattccaatacgcgatccgaatgcggcgtttcctaagcaaggtataatctcctgacgaggagtcgggtccataaggtttcca<br>tagttcaccgtgagactgcgatggtctgccaatgttcacttcaagtccgtaagacacggcaagagcctagcatctgttcgttcagagtcatggtatcggacaactgcctgatcttcgaACCCGCTGAACTTAAGCATATC<br>AATAAGCGGAGGAAAAGAAACTAACgtcacatgcaagctgtttccttctacatgacgagcctctgcgataggtgagtatcccactcattgatagctgccgcaagtcaggagaatacgtccgttagtaaactgtcccatgc<br>cgaagctcaagacctggaagtccttgataactggcacactctgagccaactgaacgtgtacgcattacaactccggtgttagcctgcttagctgaaccagcagtaattgttaggcgtcccaacgatccatgatccgcgtg<br>aagaaatctttagcgcccataggcagtaaggtagcccgacatagtgtctattaggcccgaaatcccttagggagcccaatacatgatcttagccgagtcgtaggaacgtccatctcgaaagtcgtttgctagggcaatcc<br>aagtctcgatcccgataagttctggctaggttgacaaagcgtccagatccgacgagtaaatggtccctgttaatccgatagtcgcgcaccacggtgaatatagtccgatgacattgacctgtaccagaccgcgtctcaaa<br>ttgacgaaagcgatgttcgtaaccgCGACCCGTCTTGAAACACGGACCAAGGAGTCTAAC
**Sc6001**
TGATTACGTCCCTGCCCTTTGTACACACCGCCCGTCGCTAacggcactgatgttcacccgccgtcgatcatacacgcagggcgatgactctatgcgaggctccgaccagtaacaggcgctAAACTTGGTCATTTAGAGGA<br>ACTAAAAGTCGTAACAAGGTTTCCGTAGGTGAACCTGCGGAAGGATCATTAcctggcgaatgtctaaggcgtccatatccgaggtgcagcgcgttgcctgaccattaggcccgtatagttcggcgtgaccgagatgccgc<br>tcagtacgacggtctaacaagctggccgcacttgccaacctgtcgcggactgtcttaacggtggcccgacttgctaccacacccgtgggattgtgctacgaagcgtcccgaaggtcctcagcccaagagtcctgtagtga<br>gtacccggagcctcgaccctgatgtgatccgaccagattggagccggtgaccctcagacggagtcaaggtcctacctgtgaagccctgacggcgtggattcctgctagagccaaggagagtgtcccgctacAAACTTTCA<br>ACAACGGATCTCTTGGTTCTCGCATCGATGAAGAACGCAGCGAAATGCGATACGTAATGTGAATTGCAGAATTCCGTGAATCATCGAATCTTTGAACGCACATTGCGCCCCTTGGTATTCCAGGGGGCATGCCTGTTTGA<br>GCGTCATTTgcggacgatgcctttgtcgataatgctcccgctgtaggccagcgccaatcggctgtgcatttagcgaggtctcacgccagtgcgagtacgagccttcctcctaagcgttcggtcggacaggacatctggat<br>cgcggaaccctaatcccgtgggacaccgtcacttggtcgatgcgcgtagcttgtcaccgcagggactgagaggtcaacccatgcgactgGTTTGACCTCAAATCAGGTAGGAGTACCCGCTGAACTTAAGCATATCAATA<br>AGCGGAGGAAAAGAAACCAACggtggaaagctcgtctcccaatgccattagcctcggcggagcgatagcagctcctctggaagcatcagtgcgtctgcccaaggcgttcctcgtcggtacaacgtagactgccgctacgg<br>acggtgtcaccagggatacactccatagcatccgggtcgcaaggtgtgcgtgccaactacccgacttctaacagggctggccgatactgcgggctcaagtgactcagatcctgaagggcgcaccacgtcgcggactacag<br>tgttcacatgaagcgcggtcgtgcagcgcatggtccataccaactgcctagtacgcgggactggcgtcgaatcgactcgtccttcggaaacatgacggcgcggcctaagcgagaactctgctcgtgtccatcaacggctg<br>gcggcgatatgtcctgacctcagccatagtgcctacctcgggagcgttcaagcgatcctcggtcttaacgggcgaactcgggctcgaaagcgaatgcctccctaagctcttcggtggcggacgcggaatcatagctcagc<br>gaactctcacggttgcaggcgCCGCCCGTCTTGAAACACGGACCAAGGAGTCTAAC
**Cn6001**
TGATTACGTCCCTGCCCTTTGTACACACCGCCCGTCGCTAcgtacctgtcagcacgctgttgaccttagcccgtggcaacgactgtgaagcctccgacacgtactgagggcgattcccagAAACTTGGTCATTTAGAGGA<br>AGTAAAAGTCGTAACAAGGTTTCCGTAGGTGAACCTGCGGAAGGATCAccatactgcgaatgggagccgccggaggtaagtcctttccctgatgaccttgcgcgtagggccgggtaagagcttctccactgactgtcaac<br>cgtgggcacgccgaggatgctactcatgACTTTCAACAACGGATCTCTTGGCTTCCACATCGATGAAGAACGCAGCGAAATGCGATAAGTAATGTGAATTGCAGAATTCAGTGAATCATCGAATCTTTGAACGCAACTTG<br>CGCCCTTTGGTATTCCGAAGGGCATGCCTGTTTGAGAGggcagctttacggttcccagtgcctaatgaggacgcctgggcggaatcgagccttcggaaagacatctgcagcacggtgcctgcaacctgtcggtgacgtat<br>caggacctggtgtccacccgttgtcagggcttccaaggtcaagcaagtggtgaccggccatgcgtggtcgcttcacagaacatcacggcagtcgccgtatcggcccgagtgagactagACCCGCTGAACTTAAGCATATC<br>AATAAGCGGAGGAAAAGAAACTAACgtcgtgacacgcttcgacgattgagtcgccgcctacgactgacgatcttccgcctgtagctggatgtgcccgatccgtgaggacattcccacctggactgactcgcatggagact<br>gccacggtgattcgcaacagcccgtagaggcttcgttcgaccacccgatgctgaaagctgctgcgctgatctgagacctcggagggcgtaaactggacacctgccactcggactgtgttcgcacgtcggcttcatagcca<br>ctggcaaccgcgcttgtgtgcagacggaaccctttagtgcctggcgatgaccctactcccggtgaacggcaatgcaatgggcctggaactgtgacgctcccgtaccttcccttgagaggacctggcatctggacgcaact<br>cctgggtgtgacctgtgagcaacgcctcctactgggtatagcccgcgcttagacgctgctagagccggagacatacgatccctgcgcttacacgcacgcgataggtgcgctcgataatctcggcccggtagtgcaacctg<br>accagcggtagaccttgatgacggcCGACCCGTCTTGAAACACGGACCAAGGAGTCTAAC

Table S3. Sequences of PCR primers used in this study.

Region	Primer	Sequence (5'-3')[a]	Reference[b]
V4, bacterial SSU rRNA gene	forward	GTGYCAGCMGCCGCGGTAA	Parada *et al.* (2016)
	reverse	GGACTACNVGGGTWTCTAAT	Apprill *et al.* (2015)
V9, eukaryotic/fungal SSU rRNA gene	forward	GTACACACCGCCCGTC	Lane (1991)
	reverse	TGATCCTTCYGCAGGTTCACCTAC	This study<br>Modified from Medlin *et al.* (1988)
ITS1, fungal internal transcribed spacer	forward	CTTGRTCATTTAGAGGAASTAA	This study<br>Modified from Gardes and Bruns (1993)
	reverse	GCTGCGTTCTTCATCGWTGY	This study<br>Modified from White *et al.* (1990)
ITS2, fungal internal transcribed spacer	forward	RCAWCGATGAAGAACGCAGC	This study<br>Modified from White *et al.* (1990)
	reverse	TCCTCCGCTTATTGATATGC	White *et al.* (1990)
D1D2 fungal LSU rRNA gene	forward	ACCCGCTGAACTTAAGC	Vilgalys and Hester (1990)
	reverse	GGTCCGTGTTTCAAGACGG	Kurtzman and Robnett (1997)

[a] Bases modified from the original primer sequences are underlined.

[b] References:

Parada, A.E., Needham, D.M., & Fuhrman, J.A. 2016. Every base matters: assessing small subunit rRNA primers for marine microbiomes with mock communities, time series and global field samples. Environmental Microbiology, 18(5), 1403–1414.

Apprill, A., McNally, S., Parsons, R., & Weber, L (2015. Minor revision to V4 region SSU rRNA 806R gene primer greatly increases detection of SAR11 bacterioplankton. Aquatic Microbial Ecology, 75, 129-137.

Lane, D.J. 1991. 16S/23S sequencing. In Nucleic acid technologies in bacterial systematic, eds. E. Stackebrant and M. Goodfellow. New York, New York, USA: Wiley, pp. 115–175.

Medlin, L., Elwood, H. J., Stickel, S., and Sogin, M. L. 1988. The characterization of enzymatically amplified eukaryotic 16S-like rRNA-coding regions. Gene 71, 491–499.

Gardes, M. & Bruns, T.D. 1993. ITS primers with enhanced specificity for basidiomycetes--application to the identification of mycorrhizae and rusts. Molecular Ecology 2, 113-118.

White, T.J., Bruns, T., Lee, S. & Taylor, J. 1990. Amplification and direct sequencing of fungal ribosomal RNA genes for phylogenetics. PCR Protocols: A Guide to Methods and Applications, eds Innis MA, Gelfland DH, Sninsky JJ & White TJ, eds. Academic Press, San Diego, CA, pp. 315–322.

Vilgalys, R., & Hester, M. 1990. Rapid genetic identification and mapping of enzymatically amplified ribosomal DNA from several Cryptococcus species. Journal of Bacteriology, 172, 4238-4246.

Kurtzman, C.P. & Robnett, C.J. 1007. Identification of clinically important ascomycetous yeasts based on nucleotide divergence in the 5' end of the large-subunit (26S) ribosomal DNA gene. Journal of Clinical Microbiology 35, 1216-1223.

Table S4. Summary of primer binding sites and amplicon sizes for the fungal primer sets SSU-V9, ITS1, ITS2, and LSU-D1D2. Mismatches between the primers and sequence templates are highlighted by dark red cells. Left and right blocks show the forward and reverse primers, respectively.

SSU-V9	GTACACACCGCCCGTC	TGATCCTTCYGCAGGTTCACCTAC	length
Aspergillus oryzae	GTACACACCGCCCGTC	TGATCCTTCCGCAGGTTCACCTAC	129
Candida glabrata	GTACACACCGCCCGTC	TGATCCTTCCGCAGGTTCACCTAC	129
Candida tropicalis	.......CCGCCCGTC	TGATCCTTCCGCAGGTTCACCTAC	129
Cryptococcus neoformans	GTACACACCGCCCGTC	TGATCCTTCCGCAGGTTCACCTAC	129
Emericella nidulans	GTACACACCGCCCGTC	TGATCCTTCCGCAGGTTCACCTAC	129
Hymenoscyphus varicosporoides	GTACACACCGCCCGTC	TGATCCTTCCGCAGGTTCACCTAC	129
Marasmius purpureostriatus	GTACACACCGCCCGTC	TGATCCTTCCGCAGGTTCACCTAC	129
Saccharomyces cerevisiae	.TACACACCGCCCGTC	TGATCCTTCCGCAGGTTCACCTAC	129
Schizosaccharomyces pombe	GTACACACCGCCCGTC	TGATCCTTCCGCAGGTTCACCTAC	130
Trichoderma reesei	GTACACACCGCCCGTC	TGATCC**C**TCCGC**T**GGTTCACC**A**AC	129
Cn4001	GTACACACCGCCCGTC	TGATCCTTCCGCAGGTTCACCTAC	128
Cn5001	GTACACACCGCCCGTC	TGATCCTTCCGCAGGTTCACCTAC	128
Cn5002	GTACACACCGCCCGTC	TGATCCTTCCGCAGGTTCACCTAC	128
Cn6001	GTACACACCGCCCGTC	TGATCCTTCCGCAGGTTCACCTAC	128
Sc4001	GTACACACCGCCCGTC	TGATCCTTCCGCAGGTTCACCTAC	128
Sc5001	GTACACACCGCCCGTC	TGATCCTTCCGCAGGTTCACCTAC	128
Sc5002	GTACACACCGCCCGTC	TGATCCTTCCGCAGGTTCACCTAC	128
Sc5003	GTACACACCGCCCGTC	TGATCCTTCCGCAGGTTCACCTAC	128
Sc5004	GTACACACCGCCCGTC	TGATCCTTCCGCAGGTTCACCTAC	128
Sc5005	GTACACACCGCCCGTC	TGATCCTTCCGCAGGTTCACCTAC	128
Sc5006	GTACACACCGCCCGTC	TGATCCTTCCGCAGGTTCACCTAC	128
Sc6001	GTACACACCGCCCGTC	TGATCCTTCCGCAGGTTCACCTAC	128

ITS1	CTTGRTCATTTAGAGGAASTAA	GCTGCGTTCTTCATCGWTGY	
Aspergillus oryzae	C**CC**GGTCATTTAGAGGAAGTAA	GCTGCGTTCTTCATCGATGC	257
Candida glabrata	CTTGGTCATTTAGAGGAACTAA	GCTGCGTTCTTCATCGATGC	478
Candida tropicalis	CTTGGTCATTTAGAGGAAGTAA	GCTGCGTTCTTCATCGATGC	214
Cryptococcus neoformans	CTTGGTCATTTAGAGGAAGTAA	GCTGCGTTCT**C**CATCGATGT	197
Emericella nidulans	C**CC**GGTCATTTAGAGGAAGTAA	GCTGCGTTCTTCATCGATGC	229
Hymenoscyphus varicosporoides	CTTGGTCATTTAGAGGAAGTAA	GCTGCGTTCTTCATCGATGC	242
Marasmius purpureostriatus	CTTGGTCATTTAGAGGAAGTAA	GCTGCGTTCTTCATCGATGC	295
Saccharomyces cerevisiae	CTTGGTCATTTAGAGGAACTAA	GCTGCGTTCTTCATCGATGC	437
Schizosaccharomyces pombe	CTTGGTCATTTAGAGGAAGTAA	GCTGCGTTCTTCATCGATGC	496
Trichoderma reesei	CT**C**GGTCATTTAGAGGAAGTAA	GCTGCGTTCTTCATCGATGC	296
Cn4001	CTTGGTCATTTAGAGGAAGTAA	GCTGCGTTCTTCATCGATGT	191
Cn5001	CTTGGTCATTTAGAGGAAGTAA	GCTGCGTTCTTCATCGATGT	191
Cn5002	CTTGGTCATTTAGAGGAAGTAA	GCTGCGTTCTTCATCGATGT	191
Cn6001	CTTGGTCATTTAGAGGAAGTAA	GCTGCGTTCTTCATCGATGT	191
Sc4001	CTTGGTCATTTAGAGGAACTAA	GCTGCGTTCTTCATCGATGC	436
Sc5001	CTTGGTCATTTAGAGGAACTAA	GCTGCGTTCTTCATCGATGC	436
Sc5002	CTTGGTCATTTAGAGGAACTAA	GCTGCGTTCTTCATCGATGC	436
Sc5003	CTTGGTCATTTAGAGGAACTAA	GCTGCGTTCTTCATCGATGC	436
Sc5004	CTTGGTCATTTAGAGGAACTAA	GCTGCGTTCTTCATCGATGC	436
Sc5005	CTTGGTCATTTAGAGGAACTAA	GCTGCGTTCTTCATCGATGC	436
Sc5006	CTTGGTCATTTAGAGGAACTAA	GCTGCGTTCTTCATCGATGC	436
Sc6001	CTTGGTCATTTAGAGGAACTAA	GCTGCGTTCTTCATCGATGC	436

ITS2	RCAWCGATGAAGAACGCAGC	TCCTCCGCTTATTGATATGC	
Aspergillus oryzae	GCATCGATGAAGAACGCAGC	TCCTCCGCTTATTGATATGC	314
Candida glabrata	GCATCGATGAAGAACGCAGC	TCCTCCGCTTATTGATATGC	377
Candida tropicalis	GCATCGATGAAGAACGCAGC	TCCTCCGCTTATTGATATGC	286
Cryptococcus neoformans	ACATCGATG**G**AGAACGCAGC	TCCTCCGCTTATTGATATGC	334
Emericella nidulans	GCATCGATGAAGAACGCAGC	TCCTCCGCTTATTGATATGC	313
Hymenoscyphus varicosporoides	GCATCGATGAAGAACGCAGC	TCCTCCGCTTATTGATATGC	294
Marasmius purpureostriatus	GCATCGATGAAGAACGCAGC	TCCTCCGCTTATTGATATGC	422
Saccharomyces cerevisiae	GCATCGATGAAGAACGCAGC	TCCTCCGCTTATTGATATGC	380
Schizosaccharomyces pombe	GCATCGATGAAGAACGCAGC	TCCT**G**CGCTTATTGATATGC	446
Trichoderma reesei	GCATCGATGAAGAACGCAGC	TCCTCCGCTTATTGATATGC	314
Cn4001	ACATCGATGAAGAACGCAGC	TCCTCCGCTTATTGATATGC	337
Cn5001	ACATCGATGAAGAACGCAGC	TCCTCCGCTTATTGATATGC	337
Cn5002	ACATCGATGAAGAACGCAGC	TCCTCCGCTTATTGATATGC	337
Cn6001	ACATCGATGAAGAACGCAGC	TCCTCCGCTTATTGATATGC	337
Sc4001	GCATCGATGAAGAACGCAGC	TCCTCCGCTTATTGATATGC	368
Sc5001	GCATCGATGAAGAACGCAGC	TCCTCCGCTTATTGATATGC	368
Sc5002	GCATCGATGAAGAACGCAGC	TCCTCCGCTTATTGATATGC	368
Sc5003	GCATCGATGAAGAACGCAGC	TCCTCCGCTTATTGATATGC	368
Sc5004	GCATCGATGAAGAACGCAGC	TCCTCCGCTTATTGATATGC	368
Sc5005	GCATCGATGAAGAACGCAGC	TCCTCCGCTTATTGATATGC	368
Sc5006	GCATCGATGAAGAACGCAGC	TCCTCCGCTTATTGATATGC	368
Sc6001	GCATCGATGAAGAACGCAGC	TCCTCCGCTTATTGATATGC	368

D1D2	ACCCGCTGAACTTAAGC	GGTCCGTGTTTCAAGACGG	
Aspergillus oryzae	ACCCGCTGAACTTAAGC	G**TC**C**G**GTGTTTCAAGACGG	594
Candida glabrata	ACCCGCTGAACTTAAGC	GGTCCGTGTTTCAAGACGG	603
Candida tropicalis	ACCCGCTGAACTTAAGC	GGTCCGTGTTTCAAGACGG	592
Cryptococcus neoformans	ACCCGCTGAACTTAAGC	GTCCGTGTTTCAAGACGG	621
Emericella nidulans	ACCCGCTGAACTTAAGC	GGTCCGTGTTTCAAGACGG	594
Hymenoscyphus varicosporoides	ACCCGCTGAACTTAAGC	GGTCCGTGTTTCAAGACGG	594
Marasmius purpureostriatus	ACCCGCTGAACTTAAGC	GGTCCGTGTTTCAAGACGG	622
Saccharomyces cerevisiae	ACCCGCTGAACTTAAGC	GGTCCGTGTTTCAAGACGG	594
Schizosaccharomyces pombe	AC**G**CGCTGAACTTAAGC	GGTCCGTGTTTCAAGACGG	619
Trichoderma reesei	ACCCGCTGAACTTAAGC	GGTCCGTGTTTCAAGACGG	586
Cn4001	ACCCGCTGAACTTAAGC	GGTCCGTGTTTCAAGACGG	594
Cn5001	ACCCGCTGAACTTAAGC	GGTCCGTGTTTCAAGACGG	594
Cn5002	ACCCGCTGAACTTAAGC	GGTCCGTGTTTCAAGACGG	594
Cn6001	ACCCGCTGAACTTAAGC	GGTCCGTGTTTCAAGACGG	594
Sc4001	ACCCGCTGAACTTAAGC	GGTCCGTGTTTCAAGACGG	594
Sc5001	ACCCGCTGAACTTAAGC	GGTCCGTGTTTCAAGACGG	594
Sc5002	ACCCGCTGAACTTAAGC	GGTCCGTGTTTCAAGACGG	594
Sc5003	ACCCGCTGAACTTAAGC	GGTCCGTGTTTCAAGACGG	594
Sc5004	ACCCGCTGAACTTAAGC	GGTCCGTGTTTCAAGACGG	594
Sc5005	ACCCGCTGAACTTAAGC	GGTCCGTGTTTCAAGACGG	594
Sc5006	ACCCGCTGAACTTAAGC	GGTCCGTGTTTCAAGACGG	594
Sc6001	ACCCGCTGAACTTAAGC	GGTCCGTGTTTCAAGACGG	594

Table S5. Settings for read processing using Cutadapt and DADA2's filterAndTrim function.

<table>
<tr><td>SSU-V4</td><td><pre>
cutadapt \
-g "^GTGYCAGCMGCCGCGGTAA;error_rate=2" -G "^GGACTACNVGGGTWTCTAAT;error_rate=2" \
--discard-untrimmed --max-n 0 --minimum-length 225 \
--output   R1_trimmed.fastq \
--paired-output R2_trimmed.fastq \
                R1.fastq \
                R2.fastq

dada2::filterAndTrim(fwd, rev, filt, filt.rev, truncLen = c(150,150), maxEE = c(4,4))
</pre></td></tr>
<tr><td>SSU-V9</td><td><pre>
cutadapt \
-g "^GTACACACCGCCCGTC;error_rate=2;required...GTAGGTGAACCTGCRGAAGGATCA$;error_rate=2;required" \
--discard-untrimmed --max-n 0 --minimum-length 125 \
--output R1_trimmed.fastq \
         R1.fastq

dada2::filterAndTrim(fwd, filt, maxEE = 1)
</pre></td></tr>
<tr><td>ITS1</td><td><pre>
cutadapt \
-g "^CTTGRTCATTTAGAGGAASTAA;error_rate=2;required...RCAWCGATGAAGAACGCAGC$;error_rate=2;optional" \
--discard-untrimmed --max-n 0 --minimum-length 185 \
--output R1_trimmed.fastq \
         R1.fastq

dada2::filterAndTrim(fwd, filt, maxEE = 4)
</pre></td></tr>
<tr><td>ITS2</td><td><pre>
cutadapt \
-g "^RCAWCGATGAAGAACGCAGC;error_rate=2" \
--discard-untrimmed --max-n 0 --minimum-length 225 \
--output R1_trimmed.fastq \
         R1.fastq

dada2::filterAndTrim(fwd, filt, truncLen = 225, maxEE = 4)
</pre></td></tr>
<tr><td>LSU-
D1D2</td><td><pre>
cutadapt \
-g "^ACCCGCTGAACTTAAGC;error_rate=2" -G "^GGTCCGTGTTTCAAGACGG;error_rate=2" \
--discard-untrimmed --max-n 0 --minimum-length 225 \
--output   R1_trimmed.fastq \
--paired-output R2_trimmed.fastq \
                R1.fastq \
                R2.fastq

dada2::filterAndTrim(fwd, rev, filt, filt.rev, truncLen = c(225,225), maxEE = c(6,6))
</pre></td></tr>
</table>
